# Prevalence and risk factors associated with human cystic echinococcosis in rural areas, Mongolia

**DOI:** 10.1371/journal.pone.0235399

**Published:** 2020-07-02

**Authors:** Temuulen Dorjsuren, Sumiya Ganzorig, Munkhbaatar Dagvasumberel, Altansukh Tsend-Ayush, Chimedlkhamsuren Ganbold, Mandukhai Ganbat, Enkh-Oyun Tsogzolbaatar, Uranchimeg Tsevelvaanchig, Giimaa Narantsogt, Chinchuluun Boldbaatar, Burnee Mundur, Munkhgerel Khand-Ish, Gurbadam Agvaandaram

**Affiliations:** 1 Department of Biology, Mongolian National University of Medical Sciences, Ulaanbaatar, Mongolia; 2 Laboratory of space and biological resources, National University of Mongolia, Ulaanbaatar, Mongolia; 3 Department of Radiology, Mongolian National University of Medical Sciences, Ulaanbaatar, Mongolia; 4 Department of Molecular Biology and Genetics, Mongolian National University of Medical Sciences, Ulaanbaatar, Mongolia; 5 Department of Epidemiology and Biostatistics, Mongolian National University of Medical Sciences, Ulaanbaatar, Mongolia; 6 Healthy life made possible for every citizen Non Government Organization, Ulaanbaatar, Mongolia; 7 Laboratory of helminthology, Institute of Veterinary Medicine, Ulaanbaatar, Mongolia; Taipei Medical University/Medicine, TAIWAN

## Abstract

Cystic echinococcosis is a chronic, complex and neglected zoonotic disease with considerable socio-economic impact on the affected population. Even though Mongolia is included in the list of high cystic echinococcosis risk countries, there has been very limited research and evidence on the prevalence or prevention of cystic echinococcosis. This field-based cross-sectional study to investigate the prevalence of cystic echinococcosis and its potential risk factors in Mongolia was conducted from April 2016 to March 2018. A total of 1,993 people were examined by ultrasound in five provinces of Mongolia. All cystic echinococcosis positive cases were classified according to the WHO-IWGE expert recommendations. The logistic regression model was used to detect the association between the presence of echinococcus infection and each potential risk factor. This was the first community survey based on ultrasound screening in Mongolia. We found 98 cystic echinococcosis cases (prevalence = 4.9%), including 85 abdominal ultrasound cystic echinococcosis positive cases and 13 abdominal ultrasound cystic echinococcosis negative cases (surgically treated cystic echinococcosis cases 11, and 2 confirmed cases of lung cystic echinococcosis by chestcomputed tomography in hospital of Ulaanbaatar). The prevalence of cystic echinococcosis varied greatly among different provinces, ranging from 2.0% to 13.1%. Children, elderly people and those with lower education had higher chances of getting cystic echinococcosis. Rather than dog ownership itself, daily practice for cleaning dog feces was associated with increased odds of cystic echinococcosis. The results of the present study show very high endemicity of cystic echinococcosis in Umnugovi province. Evaluation of potential risk factors associated with cystic echinococcosisshow high significance for following factors: demographics (age), social condition (education level) and hygiene practices (cleaning dog feces and use of gloves). Children under 18 and elderly people are considered as the most risk age groups in Mongolia.

## Introduction

Cystic echinococcosis (CE) is well-known as one of the most frequent zoonotic diseases in the world, and has considerable social and economic impacts on the affected human population [1–3]. According to World Health Organization (WHO) report, the incidence of CE in the world has been estimated to be over 50 cases per 100,000 people in some affected countries. The socio-economic consequences caused by CE are related with direct and indirect expenses including diagnostic procedures, hospitalization and treatment costs, and quality of human life. It has been reported as an endemic disease in many areas in the world such as Peru, Chile, Central Asia and western China. In some countries, the number of CE cases have been decreasing by using effective control programs in Iceland, New Zealand and Cyprus [4].

CE is caused by the larval stage (metacestode) of tapeworm *Echinococcus granulosus (E*.*granulosus)*. The definitive hosts of *E*.*granulosus*are various wild and domestic carnivores (Ito et al. 2013). The wild ungulate and livestock such as sheep, goat and camel are recognized as the intermediate hosts of *E*. *granulosus* [5], but humans are classified as aberrant intermediate hosts due to accidental substitution of natural hosts. Intermediate hosts are infected by *Echinococcus* eggs via ingesting contaminated food or water. The oncospheral embryos released from the eggs penetrate the small intestine of the intermediate hosts, enter the bloodstream and migrates into various organs, especially liver (80%) and lungs [6]. The oncosphere further develops into an echinococcal cyst (metacestode) which is a spherical, unilocular and fluid-filled cyst that grows gradually and contains many thousands of protoscolices.

The transmission of CE in human population is through the fecal-oral route. It has been reported that humans are mainly infected by consuming contaminated food or water, or direct contact with infected soil or dogs. Currently, many studies have confirmed that increased risk of echinococcosis infection in dogs and intermediate hosts is associated with visceral feeding, lack of anti-helmintic treatment and inadequate health education.

Mongolia is considered as a high risk country affected by CE [7]. CE cases are mostly diagnosed in referral hospitals of Ulaanbaatar, which are often at symptomatic and advanced stages of the disease and require CE cyst removal surgery. As reported by Davaatseren et al. in 1993, 18% of the surgical procedures in the First General Hospital in Ulaanbaatar, one of the three main tertiary level general hospitals in Mongolia, were done due to CE cases [8]. The recent retrospective study that reviewed in-patient records between 2008 and 2012 concluded that CE occurred in 19 provinces (out of 21) in Mongolia, but neither stage-specific diagnosis nor treatment were available [9]. The molecular biology tests of post-surgery CE samples confirmed three species of the genus *Echinococcus* transmitted in 17 provinces, namely, *E*. *granulosus*sensu stricto (G1), [10] *E*. *canadensis* (G6/7, G10), [11] and *E*. *multilocularis*(Mongolian and Asian genotypes) [12].

This study aimed to determine the prevalence of *Echinococcus* infection among the human population in Mongolia and the potential risk factors for its transmission. To our knowledge, the present study is the first study investigating the prevalence and potential risk factors associated with human CE in Mongolia. We used abdominal ultrasound for case identification and a self-administrated questionnaire to identify the potential risk factors for human CE.

## Materials and methods

### Ethics statement

Ethical approvals were obtained from: i) the Medical Ethical Review Board of Mongolian University of Medical Sciences (Approval numbers: 2015/A-002, 2017/3-05); and ii) The Research and Ethics Committee of the Ministry of Health of Mongolia (Resolution number: 2017.06.19, No-02). According to the survey approval from the Ethics Committee, written consents from all adults,12–17 years old participants and the parents of minors below 12 years were obtained.

### Study population and sampling

**Fig 1:**
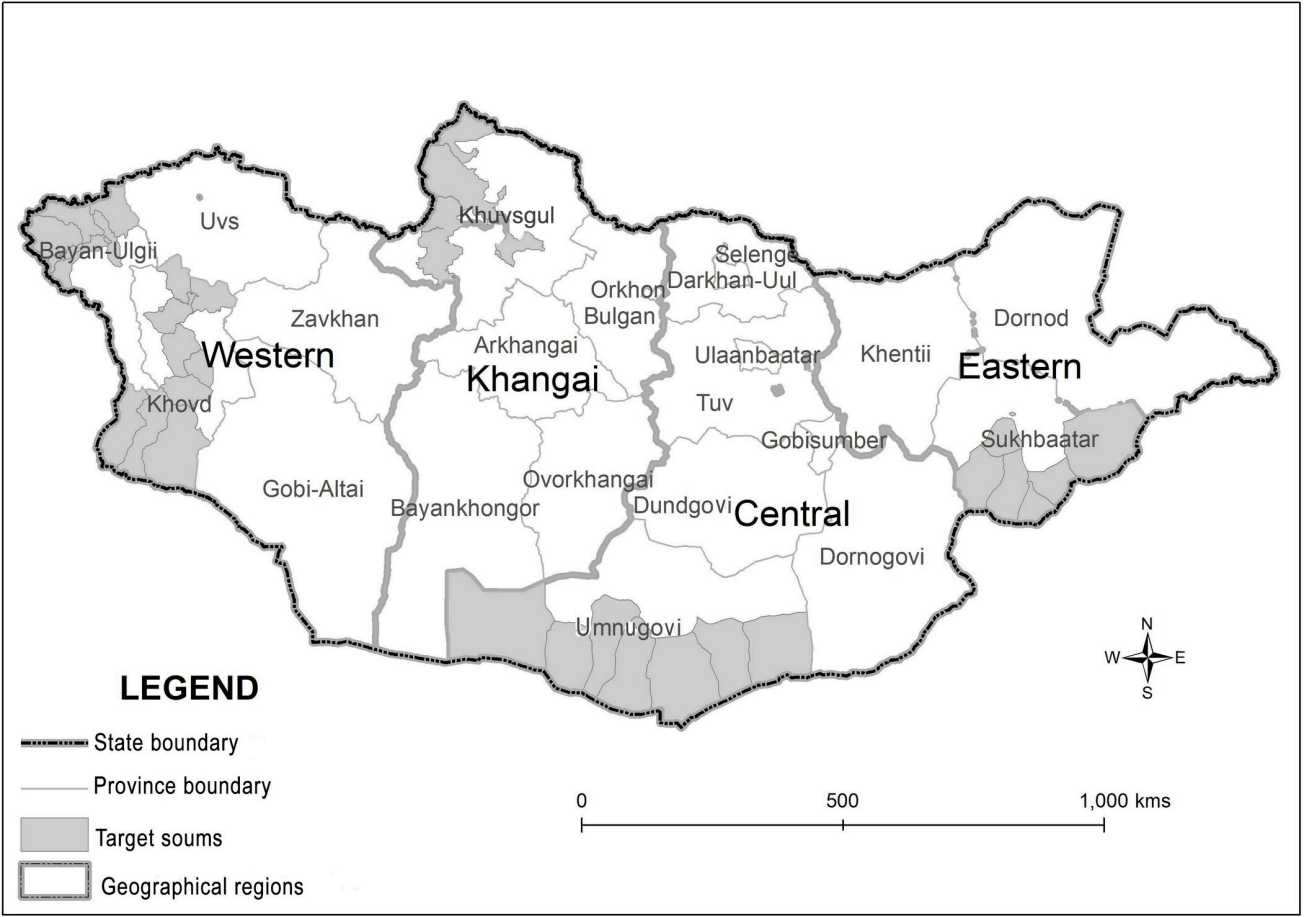
Map of Mongolia showing the study area in geographical origins. Republished from https://eic.mn/geodata/download.phpunder a CC BY license, with permission from Information and Research Institute of Meteorology, Hydrology and Environment, original copyright 2018. The figure is similar but not identical to the original image used in the study.

This was a nation-wide, a field-based cross-sectional study that involved one or two provinces randomly selected by stratified sampling design from four main geographic regions(Khovd and Bayan-Ulgii provinces from the Western region, Khuvsgul from the Northern region, Umnugovi from the Central region and Sukhbaatar from the Eastern region). 7 to 11 soums from each province, a second level administrative subdivision of Mongolia, were selected based on remoteness of each soum from the province center (to express the difficulties in getting specialized medical service), number of livestock (hypothesis risk factor) and landscape characteristics (risk factor) (Fig 1).

The sample areas were organized as follows: Western Region represents high mountainous and deserted steppe; Northern Region represents mountainous taiga; Central Region represents a desert area; and Eastern Region represents plain steppes, respectively. In total, 39 soums from 5 provinces were selected and the population size in the current study was carefully calculated in accordance to the total population of the selected provinces in 2016. The total population number of the selected provinces was 201,528 in 2016 [13]. According to the total population size, the calculated sample size in this study was 1,503 ("EpiInfoStatCalc program Sample size, U.S. Department of Health & Human Services, Accessed March 5, 2016," 2016) [14] to study the prevalence and risk factors of the infection. However, due to the large influx of peoples requesting to be examined, the actual sample size was increased and totaling 1,993 people which represents 0.99% of the total population (n = 201,528). So far, data from the 1,993 examined people was used for calculating CE prevalences. After screening, data from 1,829 questionnaires were available for the risk factor analysis. Incomplete questionnaires (51 respondents), data from 98 patients with a cystic lesion (CL), 4 patients with alveolar echinococcosis, and 11 patients who had undergone surgical removal of liver cysts were excluded. A total of 1,381 questionnaires were available for dog-related risk factor analysis. Questionnaires from non-pet participants were excluded.

### Fieldwork

The survey was conducted during five campaigns between April 2016 and March 2018. The survey participants were those who visited the Soum Health Centers (SHCs), the primary units of healthcare in Mongolia. Participants were given informed consent (adults) and informed assent (for children under 18 years old) that explained the purpose of the study, resulting in 1,993 persons officially agreeing to participate S1 File.

### Inclusion criteria

In our study, several inclusion criteria were applied to detect the risk factors-related to CE infection. It included dog owners and their family members in last 5 years, and cases with CE illness related symptoms such as the illness of the upper part of the abdomen and enlargement of abdomen.

### Exclusion criteria

To determine the risk factors related to cystic echinococcosis, some cases in this study were excluded in accordance with the following criteria. The exclusion criteria were due to any case having incomplete information on the questionnaire, presence of non-CE or cystic lesions (CL), a case with alveolar echinococcosis (AE) and CE treated cases with previous history of surgery. For dog-related risk factor analysis, the participants who had no dog at the time were excluded in further studies.

### Questionnaire

A semi-structured paper-based questionnaire **(**S2 and S3 Files) written in Mongolian was developed by our research team members. The questionnaire was taken from each participant prior to an ultrasound examination. The questions were either closed-ended or dichotomous and explanations were given when necessary. The questionnaire included demographics (gender, age, and permanent address), occupational and educational questions, and questions related to the awareness of CE, dog ownership (length of dog-ownership, feeding habits, dog feces cleaning habits, deworming routines), the at home slaughter of animals for food, and handwashing practices. The questionnaire took between 20–30 minutes to complete.

### Ultrasound

All participants (n = 1,993) who completed the questionnaire received an abdominal ultrasound (US) for the purpose of identifying of CE cases. The US was performed by experienced sonographers in Mongolia, using Mindray and Medison portable US machines, and identification of CE cases was confirmed through independent re-assessment of each lesion image review. Liver cysts were staged in accordance with the WHO-IWGE expert recommendations S4 File [3, 6]. According to the standardized US classification, the stages of CE cases were categorized into active CE (unilocular cysts or multi-vesicular with daughter vesicles), transitional CE (cysts with detachment of endocyst and predominant solid cysts with daughter vesicles) and inactive CE (Cyst solidification and calcification). CE cases diagnosed through US were advised to be referred to the National Center for Maternal and Child Health (children) and First General Hospital (adults) in the capital city.

### Serology

Serology for echinococcosis was performed using commercial *Echinococcus* IgG ELISA (Nova Lisa™, Germany) kit, according to manufacturer’s instruction S5 File. (Nova Tec) https://www.novatec-id.com/ ("Nova Tec Echinococcus IgG ELISA a guide book; Accessed 05 May 2016," 2016) [15]. For the analysis, 2 ml of venous blood was taken from all Echinococcosis (CE and AE) patients and cases with history of surgical treatment due to liver CE (n = 98). Blood samples were stored at -20°C before specific antibody testing by ELISA. The ELISA results were measured in NovaTec Units (NTU) with a cut-off of 10 NTU; 9–11 NTU was considered to be in the grey zone; those less than 9 NTU were considered negative; and results greater than 11 NTU considered positive. For the confirmation of the grey zone results, a second blood sample was taken from the patients and tested again.

### Data management and analysis

The demographic data, including gender, age, permanent address, occupation, education, living condition, producing activity with dog or livestock and hygiene practice, were analyzed by using descriptive statistics. The descriptive information is presented as mean, standard deviation (±) and frequency.

The patients with echinococcosis were applied as a dependent variable to statistically evaluate the potential risk factors-related to CE cases. Potential risk factors were carefully categorized as follows: Dog-related risk factors (dog ownership, the total number of years during which the family owned dogs, feeding of dogs raw viscera, domestic dog deworming, cleaning and destroying/eliminating dog feces, wearing gloves when you cleaning feces of a dog), social and cultural risk factors (living condition, slaughter of livestock at home, handwashing habits, education) and miscellaneous risk factors (age, gender, occupation, people`s awareness of echinococcosis).

We described the risk profile of the study population through counts for the categorical variables and through the mean and standard deviation for the continuous variables (as the distribution was normal by the Shapiro-Wilk test). A Chi-Squared test was used for the analysis of categorical variables while the Mann-Whitney U test was used for the analysis of continuous variables.

The logistic regression model was used to detect the association between the presence of CE infection and each potential risk factor. In the logistic regression model, the crude odds ratio (cOR) with 95% CI for the univariate analysis was calculated to identify the potential covariates and the adjusted odds ratio (aOR) with 95% CI for the multivariate analysis was then calculated to estimate the effects after adjustment for covariates. The Odds Ratios (OR) equal to 0 was considered as no association between outcome and exposure variable; greater than 1 indicated a risk factor and less than 1 was determined as a protective factor.

Statistical significance was set at p-value <0.05. A p-value <0.10 was considered borderline significant. Data analysis was performed using SPSS.25 (IBM Corporation, USA, 2019).

## Results

### Characteristics of the screened population

A total of 1993 people aged from 5 to 96 (mean age was 47.5±14) were examined by abdominal US. The major characteristics of the screened population presented in Table 1.

**Table 1:** Characteristics of the screened population.

Characteristics		N (%)	Mean± SD
Age (year)			47.6±14
Gender			
Male		614 (30.8)	
Female		1379 (69.2)	
**Education**			
No education and primary		232 (11.6)	
Secondary		1465(73.5)	
Tertiary		296 (14.9)	
**Occupation**			
Herders& Non-professional		1080 (54.2)	
Others		913 (45.8)	
**Living condition**			
Apartment		267 (13.4)	
Ger (traditional home)		1726 (86.6)	
**Producing activity with dogs and livestock**			
Dog ownership	Yes	1489 (74.7)	
	No	504 (25.3)	
Number of domestic dogs			1.2±0.5
Domestic dogs deworming	Yes	585 (29.4)	
	No	1408 (70.6)	
Clean and destroy/eliminate the feaces samples	Yes	1266 (63.5)	
	No	727 (36.5)	
Wear gloves when you clean feaces ofdog	Always	653 (32.7)	
	Sometimes	426 (21.4)	
	No/rarely	914 (45.9)	
Slaughter at home	Yes	1372 (68.8)	
	No	621 (31.2)	
Feeding dogs with raw viscera	Yes	1094 (54.9)	
	No	899 (45.1)	
People’s awareness of echinococcosis	Yes	615 (30.9)	
	No	1378 (69.1)	
**Hygiene practice**			
Number of hand washingsperday			4.5±3.2
Constant hand washing before meals	Yes	1642 (82.4)	
	No	351(17.6)	

The majority of participants were females (69.2%) and those with secondary education (73.5%). The participants were mainly herders and non-professionals (54.2%), and lived in gers (86.6%). The lifestyle of that category is characterized by dog ownership (74.7%), practicing at home slaughtering (68.8%) and no awareness of echinococcosis (69.1%). In each sampling area, at least 300 people were examined. The number of participants varied from a minimum of 300 in Bayan-Ulgii to maximum of 763 in Khovd Province.

### Ultrasound screening

**Fig 2:**
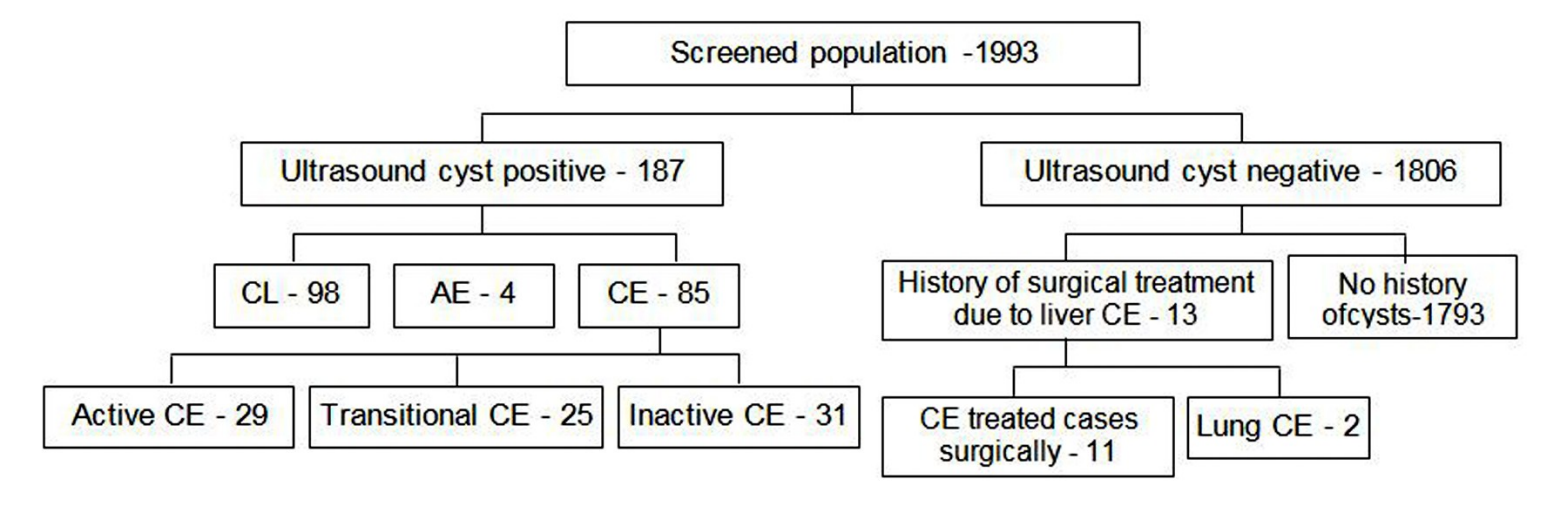
Results of ultrasound and chest computed tomography.

Out of 1993 participants, 187 were abdominal cyst positive (Fig 2). A total of 187 abdominal cyst positive cases were: i) 98 CL; ii) 4 AE; and iii) 85 CE. The majority of CE cases (85) were US positive hepatic cases, 34.1% (29) were in the active stages (CE1, CE2); 29.4% (25) were in the transitional stage (CE3a, CE3b); while 36.5% (31) were in the inactive stages (CE4, CE5) **S6 File**. In US cyst-negative cases, 13 cases were reported with previous surgical removal of cysts due to a CE diagnosis. Among them, 2 cases were diagnosed with lung CE by using chest computed tomography.

### Serology

In total, 98 blood samples, from all ultrasound *Echinococcus* positives, including 2 AE, and 2 pulmonary CE patients (diagnosed by CT) were available for *Echinococcus*IgG ELISA (Table 2). Among the 98 samples examined, 63 samples (64.3%) were antibody positive to *Echinococcus*IgG. Both pulmonary CE and one of AE samples were also positive. Only half of inactive CE cases were positive (15 from 30; 50%). However, the percentage of antibody positive was higher in samples from active CE (79.3%) and transitional CE cases (72%).

**Table 2:** Comparison of serology result and US classification.

Ultrasound results		Result of ELISA, by number (%)	
		Positive	Negative
Liver CE	Active = 29	23 (79.3)	6 (20.7)
	Transitional = 25	18 (72.0)	7 (28.0)
	Inactive = 30	15 (50.0)	15 (50.0)
	Treated = 10	4 (40.0)	6 (60.0)
LungCE = 2 (Chest CT)		2 (100.0)	0 (0.0)
Liver AE = 2		1 (50.0)	1 (50.0)
Total = 98		63(64.3)	35(35.7)

### Prevalence of human CE

The prevalence of CE in the studied provinces is presented in Table 3. The average prevalence of CE in investigated areas was 4.9%, with lowest (2.0%) in Sukhbaatar province and highest (13.1%) in Umnugovi province. Only Umnugovi province which is characterized by arid and desert land showed both the highest number of positive cases (43) and the highest prevalence compared with all other investigated areas. Prevalence’s in Western, Northern and Eastern parts of Mongolia were lower, varying from 2.0 to 4.7%. The differences in prevalence rates between Umnugovi and all other investigated provinces were statistically significant (*p<0*.*0001*).

**Table 3:** The prevalence of CE in different province.

Regions and Provinces		Number of participants	Number of CE cases	Percentage	P value*
Western	Bayan-Ulgii	300	14	4.7	<0.0001
	Khovd	763	27	3.5	
Khangai	Khuvsgul	300	8	2.7	
Central	Umnugovi	329	43	13.1	
Eastern	Sukhbaatar	301	6	2.0	
**Total**		**1993**	**98**	**4.9**	

### Potential risk factors of *Echinococcus* infection

Evaluation of 1829 questionnaires and the estimated odds ratios with p-values are presented for both univariate and multivariate models in Table 4. Potential risk factors included patient’s age, gender, level of education, occupation, living condition, knowledge of echinococcosis (awareness of echinococcosis), dog ownership, slaughtering at home, and hand washing. Univariate analysis model A (cOR) showed statistically significant (p<0.05) high risk in acquiring CE in two age groups, younger than 18 (cOR 22.73; 95% CI 9.53–54.22; p<0.0001) and older than 60 (cOR 4.68; 95% CI 2.34–9.39; p<0.0001). Low education factor (cOR 4.69; 95% CI 1.97–11.17; p<0.0005) along with dog ownership factor (cOR 0.34; 95% CI 0.22–0.53; p<0.0001) and the frequency of hand washing factor (cOR 1.80; 95% CI 1.1–2.95; p<0.019), number of hand washing per day (p<0.021) were also significant. Multivariate analysis model B (aOR) for the same potential risk factors show repeated high significance (p<0.05) for two age groups, younger than 18 (aOR 22.12; 95% CI 7.93–61.74; p<0.0001) and older than 60 (aOR 4.49; 95% CI 2.13–9.50; p<0.0001). The same significance was observed for the low education factor (aOR 5.45; 95% CI 2.11–14.07; p<0.0001). However, those in multivariate logistic regression analysis for dog ownership risk factor (aOR 0.44; 95% CI 0.24–0.77; p<0.061) and hand washing (aOR 1.33; 95% CI 0.78–2.25; p<0.289) were not supported as the previous model. But it should be noted that living in a ger (cOR 2.18; 95% CI 0.94–5.05; p<0.069) and owning a dog (aOR 0.44; 95% CI 0.24–0.77; p<0.061) are associated with borderline significance to infection.

**Table 4:** Risk factors of CE infection.

	Case n = 87	Healthy n = 1742	Total n = 1829	Model A (cOR)			Model B (aOR)		
				OR	95% CI	P value	OR	95% CI	P value
1. ***Age groups (Adjusted with gender*, *education*, *hand washing habit*, *living condition*, *dog ownership*, *awareness of CE)***									
18 or lower	15	27	42	22.73	9.53–54.22	0.0001	22.12	7.93–61.74	0.0001
19–39	11	450	461	1	-	-	1	-	-
40–59	27	968	995	1.14	0.56–2.32	0.72	1.12	0.54–2.32	0.76
60 or higher	34	297	331	4.68	2.34–9.39	0.0001	4.49	2.13–9.50	0.0001
2. Gender									
Female	57	1195	1252	1					
Male	30	547	577	1.15	0.73–1.81	0.546	-	-	-
**3. *Education (Adjusted with age*, *gender*, *living condition*, *awareness of CE*, *hand washing habit)***									
Tertiary	7	260	267	1	-	-	1	-	-
Secondary	57	1300	1357	1.63	0.73–3.61	0.23	1.92	0.82–4.51	0.134
No education or primary	23	182	205	4.69	1.97–11.17	0.0005	5.45	2.11–14.07	0.0001
4. Occupation									
Herders and Non-profession	48	950	998	1.03	0.67–1.58	0.907	-		
Professions other than herders	39	792	831	1	-	-	-	-	-
*5. Living condition*									
Apartment	6	242	248	1	-	-	-	-	-
Ger (traditional home)	81	1500	1581	2.18	0.94–5.05	0.069	-	-	-
*6. People`s awareness of echinococcosis*									
Yes	53	1206	1259	1	-	-	-	-	-
No	34	536	570	1.44	0.93–2.25	0.104	-	-	-
7. **Dog ownership (Adjusted with age, gender, education, awareness of CE, occupation, living condition, hand washing, slaughter at home)**									
Yes	46	1335	1381	0.34	0.22–0.53	0.0001	0.44	0.24–0.77	0.061
No	41	407	448	1	-	-	1	-	-
8. Slaughter at home									
Yes	55	1223	1278	0.73	0.47–1.14	0.167	-	-	-
No	32	519	551	1	-	-	-	-	-
**9. Constant hand washing before meals (Adjusted with age, gender, education, awareness of CE, dog ownership, living condition)**									
Yes	64	1452	1516	1	-		1	-	-
No	23	290	313	1.80	1.1–2.95	*0*.*019*	1.33	0.78–2.25	0.289
**10. Number of hand washing per day (Mean±SD)**									
	4.0±2.38	4.62±3.3	-	-	-	*0*.*021*^*a*^	-	-	-
^a^ Calculated by Mann-Whitney U test									

**Bold and italic:** Evidence of impact on potential infection risk by univariate and multivariate analysis.

**Bold:** Evidence of impact on potential infection risk by univariate analysis.

*Italic*: Evidence of impact on potential infection risk by univariate analysis but not significantly.

1-reference.

### Risk factors-related to *Echinococcus* infection for dog owners

The potential risk factors for transmission of CE that are related with dog ownership were analyzed by univariate and multivariate logistic regression models (Table 5). Potential risk factors included the total number of family years of having dogs, feeding dogs with raw viscera, dog deworming, cleaning the dog feces and wearing gloves during the cleaning process. In total, 1381 participants (69.3%) reportedly had a dog during the period of study. Only two variables in the study showed statistical significance for both univariate and multivariate models. Both variables belong to categories of hygienic factors such as cleaning dog feces (cOR 2.54; 95% CI 1.04–6.19; p<0.039; aOR 2.64; 95% CI 1.02–6.84; p<0.046) and using gloves during the cleaning process (cOR 2.26; 95% CI 1.12–4.53; p<0.022; aOR 2.37; 95% CI 1.17–4.88; p<0.017).

Statistically significant risk associations were not observed for the total number of family years of having dogs, feeding dogs raw viscera and dogs deworming by the univariate and multivariate regression analysis.

**Table 5:** Risk factors of CE infection for dog owners.

	Case n = 46	Healthy n = 1335	Total n = 1381	Model A (cOR)			Model B (aOR)		
				OR	95% CI	P value	OR	95% CI	P value
**1. Total number of years during the family had dogs**									
1–5 years	25	770	795	1	-	-	-	-	-
6–10 years	12	321	333	1.15	0.57–2.32	0.693	-	-	-
More than 10 years	9	244	243	1.14	0.52–2.47	0.747	-	-	-
**2. Feeding dogs with raw viscera**									
Yes	32	868	900	1.23	0.65–2.33	0.525	-	-	-
No	14	467	481	1	-	-	-	-	-
**3. Domestic dogs deworming**									
Yes	16	488	504	1	-	-	-	-	-
No	30	847	877	1.08	0.58–2.0	0.806	-	-	-
4. **Clean and destroy/eliminate the feces of dog** (Adjusted with age, gender, education, living condition, numbers of years during the family had dog, dog deworming, feeding dogs with raw viscera, wear gloves when cleaning dog feces, hand washing habit and awareness of CE)									
Burn or dispose to hole	7	279	286	1	-	-	1	-	-
Dispose waste or open hole	21	774	795	1.08	0.45–2.57	0.859	1.27	0.52–3.11	0.593
**Don`t clean responded**	**18**	**282**	**300**	**2.54**	**1.04–6.19**	**0.039**	**2.64**	**1.02–6.84**	**0.046**
5. **Wear gloves when you clean feces of dog** (Adjusted with age, gender, education, living condition, numbers of years during the family had dog, dog deworming, feeding dogs with raw viscera, clean or eliminate the feces samples, hand washing habit and awareness of CE)									
Almost all the time	13	571	584	1	-	-	1	-	-
Sometimes	11	336	347	1.44	0.64–3.25	0.382	1.58	0.66–3.79	0.303
**Rarely or no**	**22**	**428**	**450**	**2.26**	**1.12–4.53**	**0.022**	**2.37**	**1.17–4.88**	**0.017**

**Bold:** evidence of impact on potential infection risk by univariate and multivariate analysis.

1-reference.

## Discussion

In this study, we determined the prevalence and potential risk factors of human CE in five provinces of Mongolia (Bayan-Ulgii, Umnugovi, Khuvsgul, Sukhbaatar and Khovd), including 39 soums. Average prevalence of CE was 4.9%, but varied greatly depending on the particular area ranging from 2.0% to 13.1%. There were a few other community studies about CE prevalence in Mongolia [16, 17]. The screening using ELISA method involved limited number of participants in some selected areas, such as Khovd and Bayan-Ulgii [18], Tuv near Ulaanbaatar [19], Dornod and Selenge [20]. The US screening survey wasn’t available before. Prevalence rates given by those studies vary from lowest 2.1% in Dornod (4/187) to highest 11.7% (58/496) in Selenge province [20]. Previously, only Khovd and Bayan-Ulgii provinces were screened for echinococcosis. Watson-Jones et al (1997) conducted a screening of CE involving 334 herders living in Khovd and Bayan-Ulgii provinces, using the ELISA test [18]. They also estimated the CE prevalence of 5.2%, which was close to our findings (3.5% in Khovd province and 4.7% in Bayan-Ulgii province). In 2005, Wang et al. detected four CE cases in the Bulgansoum in the Khovd province [21]. Ito and Budke (2015) noted that the reliability of serology is based on the quality of the diagnostic antigens and the type of control population; and it is important to confirm cases with ultrasound and histopathology [16]. In the present study, sensitivity of IgG ELISA test was highest in active (79.3%) and transitional (72.0%) stages of CE. Lowest sensitivity was found in treated (40%) and inactive (50%) stages of CE. These differences in antibody response might also be influenced by varying origins of the pathogen. Also, Lissandrin et al. (2016) found that the serological responses assessed by commercial tests depend on CE cyst activity, and time after treatment [22].

Molecular identification of the causative species of echinococcoses in Mongolia revealed two distinct *E*. *multilocularis* genotypes, the Asian and Mongolian [16]; three different CE genotypes, *E*. *granulosuss*.*s*., *E*. *canadensis* G6/7 and *E*. *canadensis* G10 [10, 11, 23]. Temuulen et al. (2019) reported *E*. *granulosuss*.*s*.G1 and *E*. *canadensis* G6/7 from two hepatic CE patients in Western Mongolia [23]. Ito et al. (2014) for the first time examined antibody responses to rAgB in patients with CE in Mongolia. They found that nine out of 10 (90%) of *E*. *granulosuss*.*s*.G1 and 13 of 20 (65%) *E*. *canadensis* G6/7 were antibody positive [11]. Because of its diversity, accurately diagnosing and identifying the correct Echinococcus species in Mongolia is proving to be quite challenging. In this regard, combination of US, serology and molecular identification is needed.

The prevalence of CE in Mongolia is higher (see limitation of the study) in comparison with the prevalence of CE detected in western China (1.5%) [24], Morocco (1.9%) [3] and the Cusco Region of the Peruvian Highlands (3.0%) [25]. It was found to be relatively lower than in the Tibetan Plateau (6.23%) [26] and the Sichuan Province of China (6.8%) [27]. The highest CE prevalence in Mongolia was detected in Umnugovi province (13.1%). Bold et al. (2018) also found the highest number of cases in southern provinces including Umnugovi while reviewing hospital records [9]. However, older data (Galbadrah, 1972) showed that during the late 1960s in the southern regions, including Umnugovi were marked for having the lowest prevalence, 0.2 to 0.6 cases per 10,000. Therefore, the high endemicity of CE in this province should be thoroughly investigated [28]. Among the investigated areas, Umnugovi and Sukhbaatar provinces were the least populated, while Bayan-Ulgii and Khuvsgul provinces were the most populated.

Our findings of the potential risk factors for CE infection confirm to the findings of recent studies [29] and systematic reviews [30]. The highly significant potential risk factors associated with CE were found to be demographics (age), social condition (education level) and hygiene practices (cleaning dog feces and use of gloves). Age and social condition (education level) were significantly (both single and multifactor analysis) associated with CE infection. Two opposite age groups, those younger than 18 and older than 60 age, were directly associated with the CE infection. This finding was matched with many studies [30–33]. Recent studies in Mongolia (Ito et al., 2014; Bold et al., 2018) showed a high number of CE cases among children [9, 11].For instance, Ito et al (2014) analyzed 43 post-surgery cyst samples using molecular biology tests to confirm *E*. *canadensis-*infected cases. 41.9% of them (N = 18) were pediatric cases, suggesting that children have a high incidence of surgery due to of CE or other cysts [11]. Similarly, Bold et al (2018) found that 45.7% of post-surgery CE samples belonged to children [9]. Shirmen et al (2018) confirmed *Echinococcus*, in brain surgery samples taken from four children [34]. Higher CE cases among children in Mongolia indicate the necessity of public health interventions for children. Also, the lower education was quite often associated with a higher risk of CE infection [25, 29–31, 35]. In Mongolia, herders in remote areas tend to have only primary (up to year-three) and secondary level education (up to year 8 or 10). Many parents prefer to remove their children from school to get their help in animal husbandry.

Among the potential risk factors related to CE transmission, the dog owning-related factors are one of themost important factors to focus on. Statistically significant risk association was observed only for univariate analysis, but in multivariate logistic regression the dog ownership was associated with borderline significance to infection. So far, the evidence on dog owning-related risk factors for CE transmission is still uncertain and the studies produced few contradictory results. Some studies even showed no positive correlation between dog ownership and CE infections [29, 36–38] while other studies showed positive correlations [30, 31, 33]. Tamarozziet al. (2019) emphasized that CE is more “soil-transmitted” than a “food-borne” infection, acquired through a “hand-to-mouth” mechanism [3, 25, 29]. In the recent review, Possenti et al. (2016)suggested that the direct or indirect contamination of hands with *E*. *granulosuss*.*l*. eggs excreted by dogs appears to represent one of the most important pathways of transmission for human CE [30]. Result of the present survey also supports this suggestion. Both univariate and multivariate analysis models showed high significance for two hygienic factors, cleaning dog feces and use of gloves during cleaning process. These two factors are responsible for higher risk of CE infection rather than simply being a dog owner, length of dog ownership and dog deworming. The importance of wearing gloves when cleaning dog feces was also emphasized by Acosta-Jamett (2014) [39]. It was found that dog owners who clean up and destroy their dog feces were at a lower risk of acquiring infection in comparison with those who don’t clean and destroy the dog feces.

The understanding of *Echinococcus* transmission and the traditional culture of nomadic people are very crucial. Recent increase in livestock number greatly influenced spatial density of Mongolian nomad households. Closeness of households coupled with common practice of nomads to not tie dogs, inappropriate handling of dog feces supposedly increasing environmental contamination by *Echinococcus* eggs. Following studies on CE should consider impact of roaming dogs and utilization of livestock viscera. The herders are also at higher risk of *Echinococcus* transmission due to inadequate understanding of parasites and diseases. Commonly, nomads believe that the dog feces are easily eliminated in nature and there is no need to clean them up. Hence, the misunderstandings about CE and echinococcus transmission should be changed by means of healthcare control program and training to prevent the infection.

The Mongolian tradition of home slaughter of livestock for winter food stock believed to be potential risk factor, because *Echinococcus* infection is common in camels, goats, sheep and cattle in Mongolia [40, 41]. Home slaughter was not statistically significant in the present study. However, proper utilization of livestock viscera might show more value as a factor. The risk association between CE associated with home slaughter [18] and feeding the dogs with viscera [24] was found in China and other countries [30]. Also, the positive association between CE and slaughtering at home was found in Northern Chile [39] and the association between CE and the raw viscera given to dogs was observed in Florida [36], Eastern Europe and Turkey [29].

### Limitation of the study

The demographic and social condition of the sampled population does not reflect those of the local population in the investigated area. Participants of the survey were not selected; they are volunteers and supposedly those with some health problems. Thus, it has been considered that these factors might influence higher prevalence rates of CE in the present survey.

## Conclusion

The results of the present study revealed very high endemicity of CE in Umnugovi province. Evaluation of potential risk factors associated with CE shows high significance for following factors: demographics (age), social condition (education level) and hygiene practices (cleaning dog feces and use of gloves). The Umnugovi province is situated in arid and deserted area where water scarcity is very common. The present high prevalence rates over there may be related with poor hygienic practices due to lack of water. Children under 18 and elderly people are considered the most risk age groups in Mongolia. All these findings are indicating the necessity of improving CE surveillance, monitoring and measurement to prevent CE. The public-oriented information, education and communication campaigns could be targeted to increase the awareness of the population about echinococcus infection. Moreover, we suggest that the knowledge about CE prevention and infection transmission mechanisms (*hand-to-mouth*) should be included in the secondary school training program to educate the school children.

## Supporting information

S1 File Information sheet for participant and written informed participant consent. (DOCX)

S2 File Questionnaire and test for the project “The status of cystic and alveolar echinococcosis in Mongolia”, in Mongolian language. (DOCX)

S3 File Questionnaire and test for the project “The status of cystic and alveolar echinococcosis in Mongolia”, in English language. (DOCX)

S4 File Classification of liver cystic echinococcosis. (DOCX)

S5 File Protocol of echinococcusIgG-ELISA. (DOCX)

S6 File Ultrasound figures for patients with cystic echinococcosis. (PDF)
