# Supplementary material for: Prevalence and risk factors associated with human cystic echinococcosis in rural areas, Mongolia
Source: PLoS One. 2020 Jul 2;15(7):e0235399. doi: 10.1371/journal.pone.0235399 (PMC7331993; doi:10.1371/journal.pone.0235399)
Supplement: S1 File — (DOCX) [file pone.0235399.s001.docx]

**ANNEX 1**

**The status of cystic and alveolar echinococcosis**

**in Rural AREAS of Mongolia**

INFORMATION SHEET FOR Participant

(ENGLISH VERSION)

**Background and Purpose of the survey**:

Human echinococcosis is a parasitic disease caused by tapeworms of the genus *Echinococcus*. The two most important forms of the disease in humans are cystic echinococcosis (CE) and alveolar echinococcosis (AE). CE and AE result from being infected with the larval stage of *Echinococcus granulosus* (EG) complex and *Echinococcus multilocularis* (EM), respectively.

Since the collapse of centrally planned economy in 1990s, there have been increasing risk of echinococcoses in human and livestock as well as wild animal population of Mongolia. The purpose of the research is to make contribution for ceasing Echinococcus morbidity chain in some Province.

**Procedures:**

First, I will give you some information about echinococcosis. It covers life cycle of echinococcus, the way to infect humans, main symptoms of echinococcosis, prevention from the infection and its treatment.

Second, if you agree to attend the research you will be asked to have ultrasound screening. If the result of the ultrasound confirms that you have no echinococcosis you do not necessary to take a blood test. If the result of ultrasound shows that you may have echinococcosis, you will be asked to take a blood test. A small amount of blood, equal to about a teaspoon, will be taken from your arm with a syringe. This blood will be tested for the presence of echinococcus in Ulaanbaatar. The result of the blood test will be given to the Province Health Department. At the end of the research, in one year, any left over blood sample will be destroyed.

You will be also asked to answer questions about you and your behavior to contact dogs. If you have a dog, we (togheter with local veterinary service people) will visit your home and give your dog a tablet of praziquantel to treat echinococcosis and take fecal samples. After treatment the surrounding area will be disinfected. Samples will be sent to the Ulaanbaatar city for tests. Finally, if you have echinococcosis, we will give you an advise on the future treatments.

**Benefits to the patient**

If you will participate in this research, you will have the following benefits:

- your disease will be diagnosed at no charge to you
- your dog will be treated if it has infections of echinococcus other some parasits at no charge to you
- a risk of infection to your family members will be reduced

Your participation for this research will help us to decrease the prevalence of echinococcosis in your province.

**Risk from survey procedure**:

There are no clear risks associated with ultrasound and blood tests. A tablet of praziquantel kills echinococcus in the body of your dog, therefore, there are no potential risks associated with it.

**Voluntary Participation and Confidentiality of information**:

I would like to say that your participation in this research is entirely voluntary. It is your choice whether to participate or not. If you choose not to participate in this research project, it will not affect your future diagnosis and treatment in the clinic/hospital. You may change your mind later and stop participating even if you agreed earlier.

The information that we collect from this research project will be kept confidential. Information about you that will be collected during the research will be put away and no-one but the researchers will be able to see it. Any information about you will have a number on it instead of your name. Only the researchers will know what your number is and we will lock that information up with a lock and key. It will not be shared with or given to anyone except researchers.

Data files containing personal information will be stored in a locked computer with password protection at the Mongolian National University of Medical Sciences in UlaanBaatar. Access to data files will be limited the researchers**.** At the end of the study, data files containing personal identifiers will either be destroyed. You will not be personally identified when research results are published or discussed at seminars, conferences or in any other format.

**Contact Information: If you have any questions about the study,** you can contact the following persons:

| Dr. Temuulen Dorjsuren | Department of Biology and Mongolian National University of Medical Sciences, Ulaanbaatar city | Tel: (976)99170981 |
| --- | --- | --- |
| Prof. MunkhbatBatmunkh  (Chairman, Ethical Review Board) | *Director Institute of Medical Sciences,* Mongolian National University of Medical Sciences, Ulaanbaatar city | Tel: (976) 7011 1372 |

**ANNEX 2**

**The status of cystic and alveolar echinococcosis**

**in Rural AREAS of Mongolia**

**WRITTEN INFORMED Participant CONSENT**

**(ENGLISH VERSION)**

I was given the opportunity to read this information sheet. Its contents were explained and discussed with me. I was also given the chance to ask questions and I am happy with the answers I received.

_______________________ __________________________

Name of patientName of Person Giving Consent

______________________ ___________________________ __________

Name of Person Obtaining Consent Signature of Person Obtaining Consent Date of Signing

______________________

*(Mm/dd/yy)*

**WITNESS (IF NEEDED)**

This is to confirm that the information given to the potential participant above was found in the information sheet. I also heard the purpose of the survey and its procedures, the benefits and the risk it will do were discussed and explained. S/He was also given the opportunity to ask questions. S/He was also informed that s/he does not have to join the survey if s/he does not like to and that s/he can stop the interview at any time. I also witnessed the person gave her/his verbal consent to participate in the survey.

_______________ _______________________ _____________________

Name of Witness Signature of Witness Date of Signing

*(mm/dd/yy)*

**ANNEX 3**

**The status of cystic and alveolar echinococcosis**

**in Rural AREAS of Mongolia**

**INFORMATION SHEET FOR PARENTS/GUARDIANS**

ENGLISH VERSION

(Below 12 years old)

**Background and Purpose of the survey**:

Human echinococcosis is a parasitic disease caused by tapeworms of the genus *Echinococcus*. The two most important forms of the disease in humans are cystic echinococcosis (CE) and alveolar echinococcosis (AE). CE and AE result from being infected with the larval stage of *Echinococcus granulosus* (EG) complex and *Echinococcus multilocularis* (EM), respectively.

Since the collapse of centrally planned economy in 1990s, there have been increasing risk of echinococcoses in human and livestock as well as wild animal population of Mongolia. The purpose of the research is to make contribution for ceasing Echinococcus morbidity chain in some Province.

**Procedure:**

First, I will give you some information about echinococcosis. It covers life cycle of echinococcus, the way to infect humans, main symptoms of echinococcosis, prevention from the infection and treatment.

Second, should you agree for your child/children to participate in this survey s/he will be requested to have ultrasound screening. If the result of ultrasound confirms that your child has no echinococcosis your child does not necessary to take a blood test. If the result of ultrasound shows that your child may have echinococcosis, s/he will be requested to take a blood test. A small amount of blood, equal to about a teaspoon, will be taken from his/her arm with a syringe. This blood will be tested for the presence of echinococcus in Ulaanbaatar. Results of the blood test performed will be given to the Province Health Department. At the end of the research, in one year, any left over blood sample will be destroyed.

Your child also will be requested to answer questions about his/her behavior to contact dogs. If you have a dog at home, we (togheter with local veterinary service people) will visit your home and give your dog a tablet of praziquantel to treat echinococcosis and take fecal samples. After treatment the surrounding area will be disinfected. Samples will be sent to the Ulaanbaatar city for tests. Finally, if your child has an infection of echinococcus, we will give you an advise on the future treatments.

**Benefits to the Child participant**:

If your child will participate in this research, s/he will have the following benefits:

- his/her disease will be diagnosed at no charge to you
- your dog will be treated if it has infections of echinococcus other some parasites at no charge to you
- a risk of infection to your family members will be reduced

Participation of your child for this research helps us to decrease the prevalence of echinococcosis in your province.

**Risk from survey procedure**:

There are no clear risks associated with ultrasound and blood tests. Tablet of praziquantel kills echinococcus in your dog, therefore, there are no potential risks associated with it

**Voluntary Participation and Confidentiality of information**:

Your decision to have your child participate in this study is entirely voluntary. It is your choice whether to have your child participate or not. You may also choose to change your mind later and stop participating, even if you agreed earlier, that is no problem.

The information that we collect from this research project will be kept confidential. Information about your child that will be collected during the research will be put away and no-one but the researchers will be able to see it. Any information about your child will have a number on it instead of his/her name. Only the researchers will know what your child number is and we will lock that information up with a lock and key. It will not be shared with or given to anyone except researchers.

Data files containing personal information will be stored in a locked computer with password protection at the Mongolian National University of Medical Sciences in Ulaanbaatar. Access to data files will be limited to the researchers only**.** At the end of the study, data files containing personal identifiers will either be destroyed. Your child will not be personally identified when research results are published or discussed at seminars, conferences or in any other format.

**Contact Information: If you have any questions about the study,** you can contact the following persons:

| Dr. Temuulen Dorjsuren | Department of Biology,Mongolian National University of Medical Sciences, Ulaanbaatar city | Tel: (976) 99170981 |
| --- | --- | --- |
| Prof. MunkhbatBatmunkh (Chairman, Ethical Review Board | *Director Institute of Medical Sciences,* Mongolian National University of Medical Sciences, Ulaanbaatar city | Tel: (976) 7011 1372 |

**ANNEX 4**

**The status of cystic and alveolar echinococcosis**

**in Rural AREAS of Mongolia**

**WRITTEN INFORMED PARENTAL CONSENT**

**(ENGLISH VERSION)**

(Below 12 years old)

I was given the opportunity to read this information sheet. Its contents were explained and discussed with me. I understand that my child’s participation in this study is voluntary. I was also given the chance to ask questions and I am happy with the answers I received. After due consideration, I give consent for my child to join in this survey.

__________________________ _______________________

Name of Child Name of Person Giving Consent Relationship to Child

_______________________ __________________________

Signature or Thumbprint of Date of signing *(mm/dd/yy)*

Parents/Guardians

______________________ ___________________________

Name of Person Obtaining Consent Signature of Person Obtaining Consent

______________________

Date of signing*(mm/dd/yy)*

**WITNESS (IF NEEDED)**

This is to confirm that the information given to the potential participant above was found in the information sheet. I also heard the purpose of the survey and its procedures, the benefits and the risk it will do were discussed and explained. S/He was also given the opportunity to ask questions. S/He was also informed that s/he does not have to join the survey if s/he does not like to and that s/he can stop the interview at any time. I also witnessed the person gave her/his verbal consent to participate in the survey.

_______________________ _____________________

Name of Witness Signature of Witness

______________

Date of signing *(mm/dd/yy)*

**ANNEX 5**

**The status of cystic and alveolar echinococcosis**

**in Rural AREAS of Mongolia**

**INFORMATION SHEET FOR CHILDREN AGE 12-17 YEARS OLD**

(ENGLISH VERSION)

**Background and Purpose of the survey**:

Human echinococcosis is a parasitic disease caused by tapeworms of the genus *Echinococcus*. The two most important forms of the disease in humans are cystic echinococcosis (CE) and alveolar echinococcosis (AE). CE and AE result from being infected with the larval stage of *Echinococcus granulosus*(EG) complex and *Echinococcus multilocularis*(EM), respectively.

Since the collapse of centrally planned economy in 1990s, there have been increasing risk of echinococcoses in human and livestock as well as wild animal population of Mongolia. The purpose of the research is to make contribution for ceasing Echinococcus morbidity chain in some Province.

**Procedures:**

First, I will give you some information about echinococcosis. It covers life cycle of echinococcus, the way to infect humans, main symptoms of echinococcosis, prevention from the infection and treatment.

Second, should you agree to participate in this survey you will be requested to have ultrasound screening. If the result of ultrasound confirms that you have no echinococcosis you do not necessary to take a blood test. If the result of ultrasound shows that you may have echinococcosis, you will be requested to take a blood test. A small amount of blood, equal to about a teaspoon, will be taken from your arm with a syringe. This blood will be tested for the presence of echinococcus in Ulaanbaatar. Results of the blood test performed will be given to the Province Health Department. At the end of the research, in one year, any left over blood sample will be destroyed.

You will be also requested to answer questions about your behavior to contact dogs. If you have a dog at home, we (togheter with local veterinary service people) will visit your home and give your dog a tablet of praziquantel to treat echinococcosis and take fecal samples. After treatment the surrounding area will be disinfected. Samples will be sent to the Ulaanbaatar city for tests. Finally, if you have an infection of echinococcus, we will give you an advise on the future treatments.

**Benefits to the Child participant**:

If you participate in this research, you will have the following benefits:

- your disease will be diagnosed at no charge to you
- your dog will be treated if your dog has an infection at no charge to you
- this research helps to prevent your family members from this infection in the future
- you will be aware of the parasites, which are transferred from pets to humans and makes people sick and the ways to prevent from this infectious disease.

**Risk from survey procedure**:

Both ultrasound and blood tests are safe. Many people have these procedures quite often without any danger. You do not have to be worry. The tablet, which we are going to give your dog, kills parasites in the body of your dog, therefore, this tablet will help your dog to become healthy.

**Voluntary Participation and Confidentiality of information**:

You don't have to be in this research if you don't want to be. It’s up to you. If you decide not to be in the research, it’s okay. Even if you say "yes" now, you can change your mind later and it’s still okay.

We will not tell other people that you are in this research and we won't share information about you to anyone who does not work in the research study.Onlyyou and your parents will be told the results of the ultrasound and blood tests.

Information about you that will be collected from the research will be put away and no-one but the researchers will be able to see it. Any information about you will have a number on it instead of your name. Only the researchers will know what your number is and we will lock that information up with a lock and key.

Data files containing personal information will be stored in a locked computer with password protection at the Mongolian National University of Medical Sciences in UlaanBaatar. Access to data files will be limited to researchers of this study**.** At the end of the study, data files containing personal identifiers will either be destroyed. You will not be personally identified when research results are published or discussed at seminars, conferences or in any other format.

**Contact Information: If you have any questions about the study,** you can contact the following persons:

| Dr. Temuulen Dorjsuren | Department of Biology, Mongolian National University of Medical Sciences, Ulaanbaatar city | Tel: (976) 99170981 |
| --- | --- | --- |
| Prof. MunkhbatBatmunkh  (Chairman, Ethical Review Board) | *Director Institute of Medical Sciences,* Mongolian National University of Medical Sciences, Ulaanbaatar city | Tel: (976) 7011 1372 |

**ANNEX 6**

**The status of cystic and alveolar echinococcosis**

**in Rural AREAS of Mongolia**

**ASSENT FORM (ENGLISH VERSION)**

(12-17 years old)

The above information has been discussed with me in detail and has been understood by me. I was given opportunity to ask questions and was satisfied with answers given. My participation in this project is voluntary and I can withdraw anytime without prejudice to any medical treatment that I ought to receive. My signature below signifies my consent to participate in this study.

_______________________ __________________________

Name of Child Signature of Child

___________________

Date of signing*(mm/dd/yy)*

___________________ __________________________

Name of Person Obtaining Consent Signature of Person Obtaining Consent

Date of signing*(mm/dd/yy)*
