# Supplementary material for: Prevalence and risk factors associated with human cystic echinococcosis in rural areas, Mongolia
Source: PLoS One. 2020 Jul 2;15(7):e0235399. doi: 10.1371/journal.pone.0235399 (PMC7331993; doi:10.1371/journal.pone.0235399)
Supplement: S2 File — (DOCX) [file pone.0235399.s002.docx]

S1 Appendix

**“Бэтэг өвчний байгаль дах гинжин хэлхээг сааруулах нь”**

**төсөлд оролцогсодоос авах асуумж**

**Бид таны хувийн мэдээлэл болон хариултыг чандлан хадгалах болно.**

**(Таны хариулт зөвхөн судалгааны зориултаар ашиглагдана)**

Судлагааг үнэн зөв бөглөнө үү.

Судлагаанд оролцогчдийн код____________________

| **№** | **Асуулт** | **Хариулт** |
| --- | --- | --- |
|  | Нэр |  |
|  | Овог |  |
|  | Төрсөн он, сар, өдөр |  |
|  | Нас (жилээр) |  |
|  | Хүйс | Эрэгтэй  Эмэгтэй |
|  | Жин |  |
|  | Холбоо барих мэдээлэл   - Суурин хаяг - Холбоо барих утас |  |
|  | Боловсрол | Дээд  Тусгай дунд  Бүрэн дунд  Бүрэн бус дунд  Бага Боловсролгүй |
|  | Ажил мэргэжил |  |
|  | Орон байрны нөхцөл | Гэр хороолол  Орон сууц |
|  | Гэрлэлтийн байдал | Гэрлэсэн  Бэлэвсэн  Салсан  Ганц бие |
| 12. | Та нохой тэжээдэг үү? | Тийм  Үгүй |
| 13. | Хэрэв тийм бол хэдэн жил нохой тэжээж байгаа вэ? | 1-5 жил  6-10 жил  10-аас илүү жил |
| 14. | Таны нохой танай гэрт амьдардаг уу? | Тийм  Үгүй |
| 15. | Та нохойтойгоо хэрхэн харилцдаг вэ? |  |
| 16. | Таны нохой хараа хяналтгүй гадагшаагаа гардаг уу? | Тийм  Үгүй |
| 17. | Та гэрийн тэжээвэр нохойгоо 3 сар тутамд ариутгадаг уу? | Тийм  Үгүй |
| 18. | Та үнэг эсвэл зэрлэг нохой агнадаг уу? | Тийм  Үгүй |
| 19. | Хэрэв тийм бол жилд хэдэн удаа? |  |
| 20. | Та гэрийн нөхцөлд мал нядалдаг уу? | Тийм  Үгүй |
| 21. | Хэрэв тийм бол та нохойгоо малын түүхий, шүүрхий дотор эрхтнээр хооллодог уу? | Тийм  Үгүй |
| 22. | Та нохойныхоо баасыг хаана, яаж устгадаг вэ? | Шатаах эсвэл нүх рүү хаях  Хог хаягдлаар хаях эсвэл ил хаях  Цэвэрлэдэггүй |
| 23. | Та нохойныхоо баасыг цэвэрлэхдээ бээлий өмсдөг үү? | Байнга  Ихэвчлэн  Заримдаа  Хааяа  Үгүй |
| 24. | Та юм идэхийн өмнө гараа тогтмол угаадаг уу? | Тийм  Үгүй |
| 25. | Та гараа өдөрт хэдэн удаа угаадаг вэ? | 1-2 3-4 4-6 |
| 26. | Та түүхий ногоо иддэг үү? | Тийм  Үгүй |
| 27. | Таны (гэр бүлийн) ундны усны эх үүсвэр юу вэ? |  |
| 28. | Нохойноос хүнд халдварладаг өвчнүүдийг нэрлэнэ үү? | Боом  Галзуу  Тарваган тахал  **Бруцеллёкз**  Томуу  Сүрьеэ  Шүлхий  Бэтэг |
| 29. | Та хүн малын Бэтэг өвчний талаар мэдэх үү ? | Тийм  Үгүй |
| 30. | Бэтэг өвчин ямар замаар нохойноос хүн рүү халдварлах вэ? | Агаар дусал  Амаар  Арьс шархаар  Бусад |
| 31. | Бэтэг өвчин халдварлахаас хэрхэн урьдчилан сэргийлэх вэ? | Хувийн ариун цэвэр  Хувийн ариун цэвэр болон мал эмнэлэгийн үйлчилгээ  Мал эмнэлэгийн үйлчилгээ  Мэдэхгүй |
| 32. | Та Бэтэг өвчинөөр өвчилж байсан уу? | Тийм  Үгүй |
| 33. | Хэрэв тийм бол мэс засалд орж байсан эсэх? | Тийм  Үгүй |
| 34. | Бэтэг өвчиний улмаас хэдэн удаа мэс засалд орж байсан бэ? | 1  2  3  4  5-аас илүү |
| 35. | Уг өвчлөл анх хэзээ, хаана оношлогдсон бэ? |  |
| 36. | Эмчлэгдсэн эсэх? | Тийм  Үгүй |
| 37. | Та хэзээ, хаана хамгийн сүүлд Бэтэг өвчиний шинжилгээ өгсөн бэ? |  |
| 38. | Эмнэлзүйн шинж: танд ямар нэгэн зовиур байгаа бол бичнэ үү? | Хэвлийн дээд хэсгийн өвдөлт  Хэвлийн өвдөлт  Бусад |
