# Supplementary material for: Prevalence and risk factors associated with human cystic echinococcosis in rural areas, Mongolia
Source: PLoS One. 2020 Jul 2;15(7):e0235399. doi: 10.1371/journal.pone.0235399 (PMC7331993; doi:10.1371/journal.pone.0235399)
Supplement: S3 File — (DOCX) [file pone.0235399.s003.docx]

S1 Appendix

**Questionnaire and test for the project**

**“The status of cystic and alveolar echinococcosis**

**in Mongolia”**

**We will strictly protect your response and information.**

**(We will only use for research purposes)**

Please. If you answer sincerely, you will help us for implement this project

Code of survey participant____________________

| **№** | **Question** | **Answer** |
| --- | --- | --- |
|  | First name |  |
|  | Family name |  |
|  | Date of birth |  |
|  | Age (by year) |  |
|  | Gender | Male  Female |
|  | Weight |  |
|  | Contact details   - Permanent address - Phone number |  |
|  | Education | Tertiary Secondary Primary No education |
|  | Occupation |  |
|  | Living condition | Ger (traditional home)  Apartment |
|  | Marriage status | MarriedDowagerDivorced Never married |
| 12. | Do you have a dog? | Yes  No |
| 13. | If yes, how long have you had a dog? | 1-5 years6-10 years More than 10 years |
| 14. | Is your dog live within your house or not? | Yes  No |
| 15. | How do you communicate with your dog? |  |
| 16. | Does your dog go outdoors without your attention? | Yes  No |
| 17. | Do you deworming your domestic dog for every 3 months? | Yes  No |
| 18. | Do you hunt foxes or wild dogs? | Yes  No |
| 19. | If yes, how often per year? |  |
| 20. | Do you slaughter the cattle by yourself at your home? | Yes  No |
| 21. | If yes, do you feed your dog with raw viscera? | Yes  No |
| 22. | How do you clean and destroy/eliminate the feces of your dog? | Burn or dispose to hole Dispose waste or open hole  Don`t clean |
| 23. | Do you wear gloves when you clean feces of your dog? | Always Often  Sometimes  Rarely  No |
| 24. | Do you wash your hands constantly before meals? | Yes  No |
| 25. | How many times do you wash your hands for per day? | 1-2 3-4 4-6 |
| 26. | Do you eat raw vegetables? | Yes  No |
| 27. | What is the source of your (family) drinking water? |  |
| 28. | Which infection is transmitted from dog to humans? | Cowpox Rabies Canine distemper  **Foot**-**and**-**mouth** **disease**Echinococcosis  Tuberculosis |
| 29. | Do you know about echinococcosis? | Yes  No |
| 30. | How echinococcosis transmitted from dog to humans? | through the air hand-to-mouth  through skin other |
| 31. | How would you prevent from echinococcosis? | hygiene,  hygiene and service veterinary,  service veterinary, don’t know |
| 32. | History of echinococcosis? | Yes  No |
| 33. | History of surgery due to echinococcosis? | Yes  No |
| 34. | If yes, how many times did you have surgery due to echinococcosis? | 1  2 3  4  more than 5 |
| 35. | When and where your echinococcosis was diagnosed at the first time? |  |
| 36. | Have you had a treatment? | Yes  No |
| 37. | When and where was your last diagnostic test of echinococcosis? |  |
| 38. | Do you have any symptoms of echinococcosis? | Pain in the upper abdomen  Larger abdomen  Others |

**Thank you for your support**
