# Supplementary material for: Prevalence and risk factors associated with human cystic echinococcosis in rural areas, Mongolia
Source: PLoS One. 2020 Jul 2;15(7):e0235399. doi: 10.1371/journal.pone.0235399 (PMC7331993; doi:10.1371/journal.pone.0235399)
Supplement: S4 File — (DOCX) [file pone.0235399.s004.docx]

**Classification of liver cystic echinococcosis**

<https://radiopaedia.org/articles/2001-who-classification-of-hepatic-hydatid-cysts>

| **CL**   - - unilocular anechoic cystic lesion   - no any internal echoes or septations | 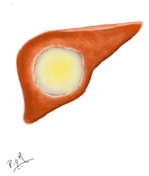 |
| --- | --- |
|  | Figure 1 |
| **CE 1**(active stage)   - - uniformly anechoic cyst with fine internal echoes, may only be visible after patient repositioning ^2^   - internal echoes represent "hydatid sand" (fluid and protoscolices originating from a ruptured vesicle) ^2^ | 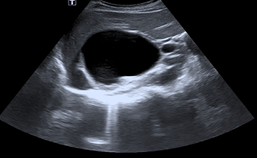 |
|  | Case1 |
| **CE 2**(active stage)   - - cyst with internal septation     - septa represent walls of daughter cyst(s) ^2^     - described as multivesicular, rosette, or honeycomb appearance | 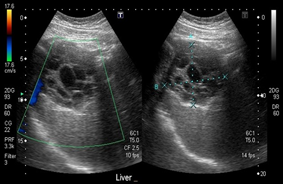 |
|  | Case 2 |
| **CE 3**(transitional stage)   - - evolving appearance of daughter cyst(s) within the encompassing parent cyst   - **3A** - daughter cysts have detached laminated membranes ([water lily sign](https://radiopaedia.org/articles/water-lily-sign-hydatid-cyst?lang=us)) | 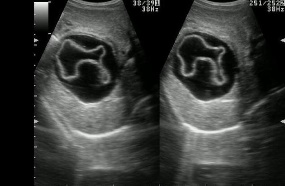 |
|  | Case 3 |
| - - **3B** - daughter cysts within a solid matrix | 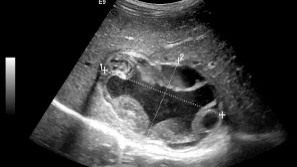 |
|  | Case 4 |
| **CE 4**(inactive/degenerative)   - - absence of daughter cysts   - mixed hypoechoic and hyperechoic matrix, resembling a ball of wool ([ball of wool sign](https://radiopaedia.org/articles/ball-of-wool-sign-hydatid-cyst?lang=us)) | 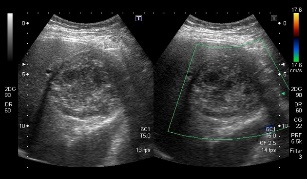 |
|  | Case 5 |
| - **CE 5**(inactive/degenerative)   - arch-like, thick partially or completely calcified wall | 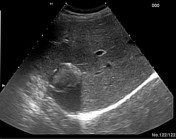 |
|  | Case 6 |

**Abbreviations**

CL: cystic lesion, CE: cystic echinococcosis

## References

- 1. Giorgio A, Di Sarno A, de Stefano G et-al. Sonography and clinical outcome of viable hydatid liver cysts treated with double percutaneous aspiration and ethanol injection as first-line therapy: efficacy and long-term follow-up. AJR Am J Roentgenol. 2009;193 (3): W186-92. [doi:10.2214/AJR.08.1518](http://dx.doi.org/10.2214/AJR.08.1518) - [Pubmed citation](http://www.ncbi.nlm.nih.gov/pubmed/19696257)
- 2. Pakala T, Molina M, Wu GY. Hepatic Echinococcal Cysts: A Review. (2016) Journal of clinical and translational hepatology. 4 (1): 39-46. [doi:10.14218/JCTH.2015.00036](https://doi.org/10.14218/JCTH.2015.00036) - [Pubmed](https://www.ncbi.nlm.nih.gov/pubmed/27047771)
