# Supplementary material for: Prevalence and risk factors associated with human cystic echinococcosis in rural areas, Mongolia
Source: PLoS One. 2020 Jul 2;15(7):e0235399. doi: 10.1371/journal.pone.0235399 (PMC7331993; doi:10.1371/journal.pone.0235399)
Supplement: S5 File — (DOCX) [file pone.0235399.s005.docx]

**Protocol of Echinococcus IgG-ELISA**

1. Dispense 100µl controls and diluted samples into their respective wells. Leave well A1 for substrate blank.
2. Cover wells with the foil supplied in the kit.
3. **Incubate for 1 hour ± 5 min at 37±1°C.**
4. When incubation has been completed, remove the foil, aspirate the content of the wells and wash each well three times with 300 µl of Washing Solution. Avoid overflows from the reaction wells. The soak time between each wash cycle should be >5 sec. At the end carefully remove remaining fluid by tapping strips on tissue paper prior to the next step!

*Note: Washing is critical! Insufficient washing results in poor precision and falsely elevated absorbance values.*

1. Dispense 100 µl Echinococcus anti-IgG conjugate into all wells except for the blank well (e.g. A1). Cover with foil.
2. **Incubate for 30 min at room temperature**. Do not expose to direct sunlight.
3. Repeat step 4.
4. Dispense 100 µl TMB Substrate Solution into all wells
5. **Incubate for exactly 15 min at room temperature in the dark.**
6. Dispense 100 µl stop solution into all wells in the same order and at the same rate as for the TMB Substrate Solution.

*Any blue colour developed during the incubation turns into yellow.*

*Note: Highly positive patient samples can cause dark precipitates of the chromogen! These precipitates have an influence when reading the optical density. Predilution of the sample with physiological sodium chloride solution, for example 1+1, is recommended. Then dilute the sample 1+100 with dilution buffer and multiply the results in NTU by 2.*

1. Measure the absorbance of the specimen at 450/620 nm within 30 min after addition of the Stop Solution.

**Measurement**

Adjust the ELISA Microwell Plate Reader to **zero** using the **substrate blank in well A1**.

If - due to technical reasons - the ELISA reader cannot be adjusted to zero using the substrate blank

in well A1, subtract the absorbance value of well A1 from all other absorbance values measured in order to obtain reliable results!

**Measure the absorbance** of all wells at **450 nm** and record the absorbance values for each control and patient sample in the distribution and identification plan.

*Dual wavelength reading using 620 nm as reference wavelength is recommended.*

Where applicable calculate the **mean absorbance** **values** of all duplicates.

**Run Validation Criteria**

In order for an assay to be considered valid, the following criteria must be met:

- **Substrate blank** in A1: Absorbance value < **0.100.**
- **Negative control** in B1: Absorbance value < **0.200 and < cut-off**
- **Cut-off control**  in C1 and D1: Absorbance value **0.150 – 1.30.**
- **Positive control** in E1: Absorbance value > **cut-off**.

If these criteria are not met, the test is not valid and must be repeated.

**Calculation of Results**

The cut-off is the mean absorbance value of the Cut-off control determinations.

*Example*: Absorbance value Cut-off control 0.54 + absorbance value Cut-off control 0.52 =1.06 / 2 = 0.53

Cut-off = 0.53

**Interpretation of Results**

Samples are considered POSITIVE if the absorbance value is higher than 10% over the cut-off.

Samples with an absorbance value of 10% above or below the cut-off should not be considered as clearly positive or negative

- **grey zone**

It is recommended to repeat the test again 2 - 4 weeks later with a fresh sample. If results in the second test are again in the grey zone the sample has to be considered **NEGATIVE**.

Samples are considered **NEGATIVE** if the absorbance value is lower than 10% below the cut-off.

**Results in Nova Tec Units**

*Example:* $\frac{1.786 x 10}{0.53}= 34 NTU (NovaTec Units)$

Cut-off: 10 NTU

Grey zone: 9-11 NTU

Negative: <9 NTU

Positive: >11 NTU
