# Supplementary material for: Prevalence and risk factors associated with human cystic echinococcosis in rural areas, Mongolia
Source: PLoS One. 2020 Jul 2;15(7):e0235399. doi: 10.1371/journal.pone.0235399 (PMC7331993; doi:10.1371/journal.pone.0235399)
Supplement: S6 File — (PDF) [file pone.0235399.s006.pdf]

ULTRASOUND FIGURES IN PATIENTS OF BAYAN-ULGII PROVINCE

Patient 1
Male, 11 age

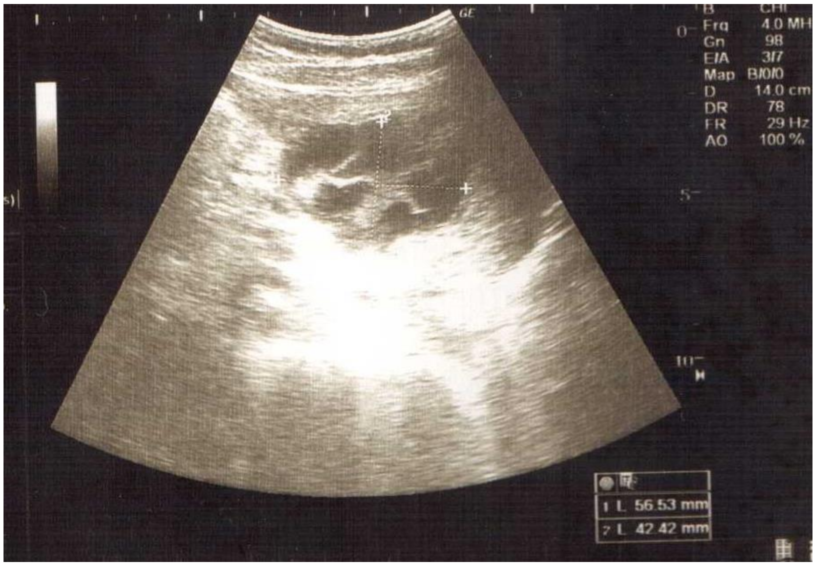

Figure 1: 5.6x4.2 cm, anechoic, univesicular cystic
with detached layers in the liver. WHO's classification – CE3.

Patient 2
Female, 17age

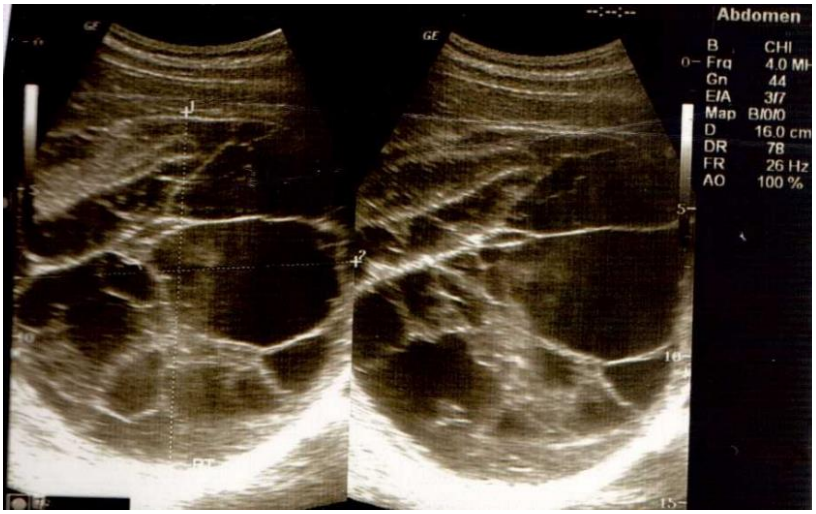

Figure 2: Well defined, multiseptated, anechoic cyst /with
double wall sign/ in the liver. WHO's classification – CE2.

Patient 3

Female, 67 age

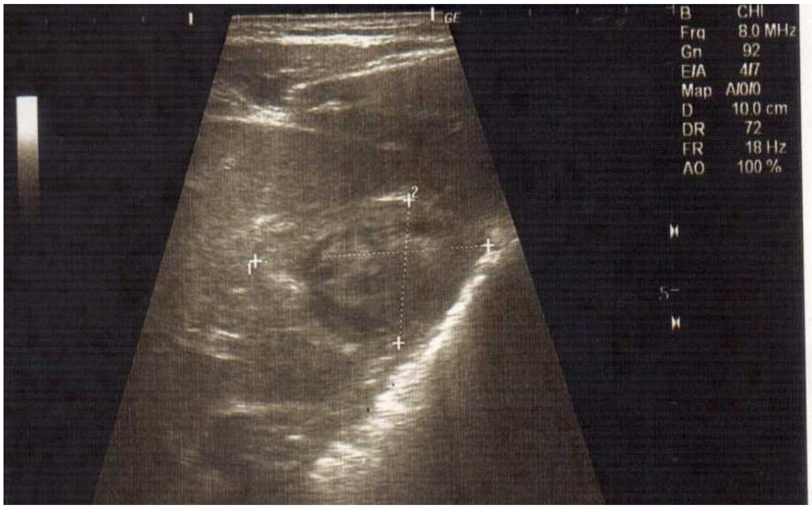

Figure 3: Heterogeneous /hyper and hypo/ echoic mass /with acoustic shadow/ in the liver. WHO's classification – CE4

Patient 4

Female, 32 age

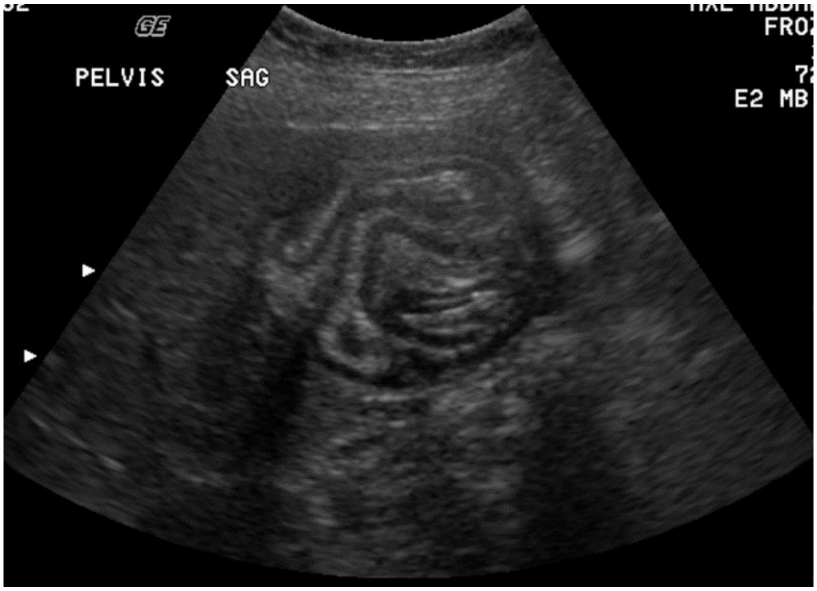

Figure 4: Heterogeneous /hyper and hypo/ echoic mass /with acoustic shadow/ in the liver. WHO's classification – CE4

Patient 5

Female, 61 age

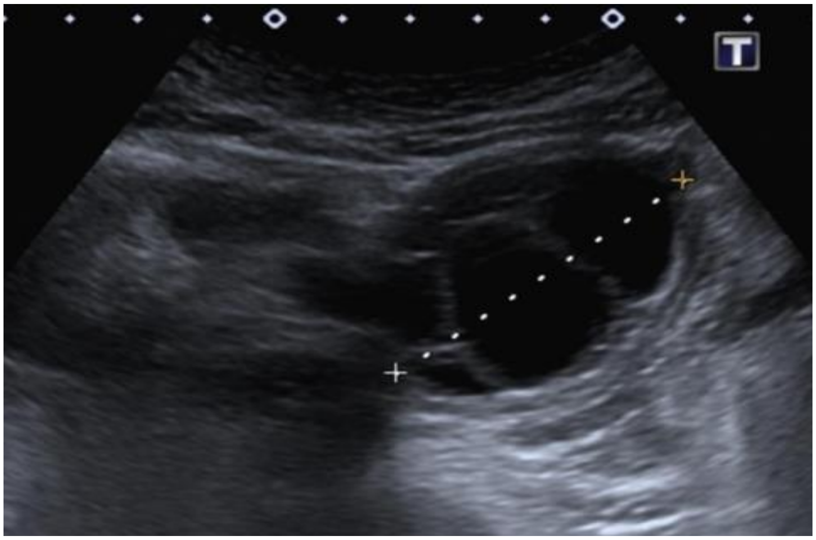

Figure 5: Well defined, multiseptated, anechoic cyst /with double wall sign/ in the liver. WHO’s classification – CE2.

Patient 6

Female, 15age

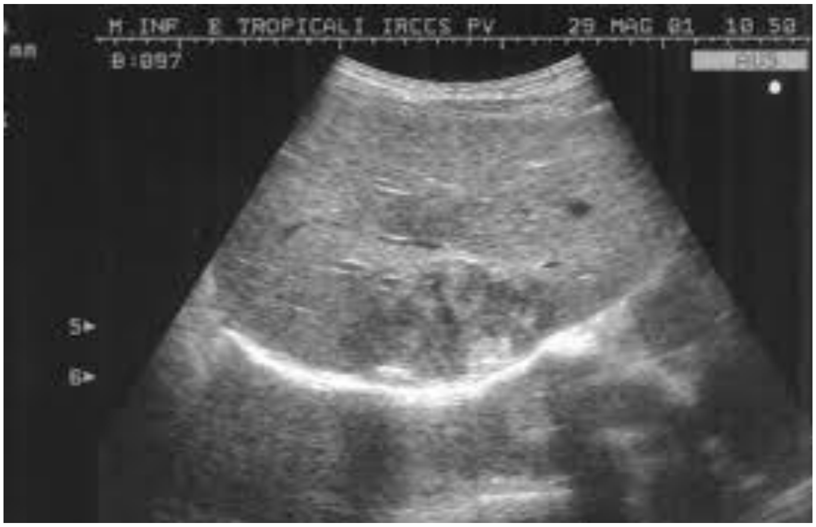

Figure 6: Heterogeneous /hyper and hypo/ echoic mass /with acoustic shadow/ in the liver. WHO’s classification – CE4

Patient 7
Female, 31age

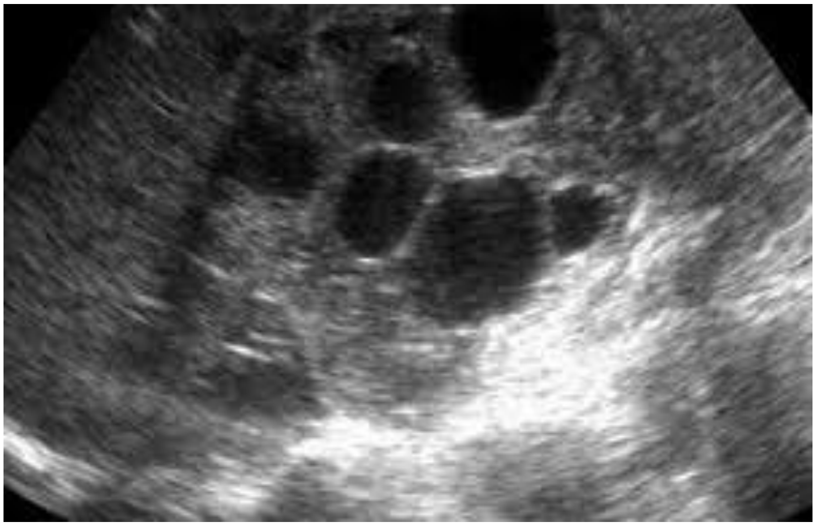

Figure 7: Well defined, multiseptated, anechoic cyst /with double wall sign/ in the liver. WHO’s classification – CE2.

Patient 8
Female, 17age

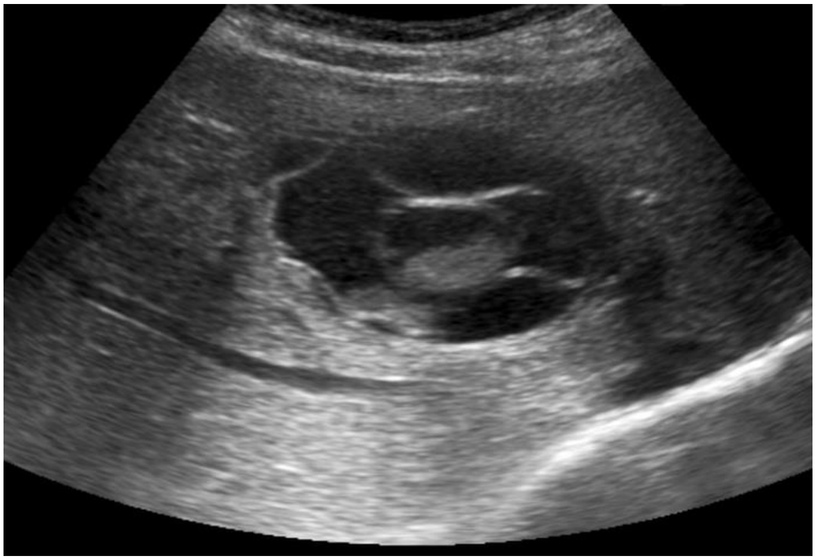

Figure 8: Well defined, multiseptated, anechoic cyst /with double wall sign/ in the liver. WHO’s classification – CE2.

Patient 9

Female, 60 age

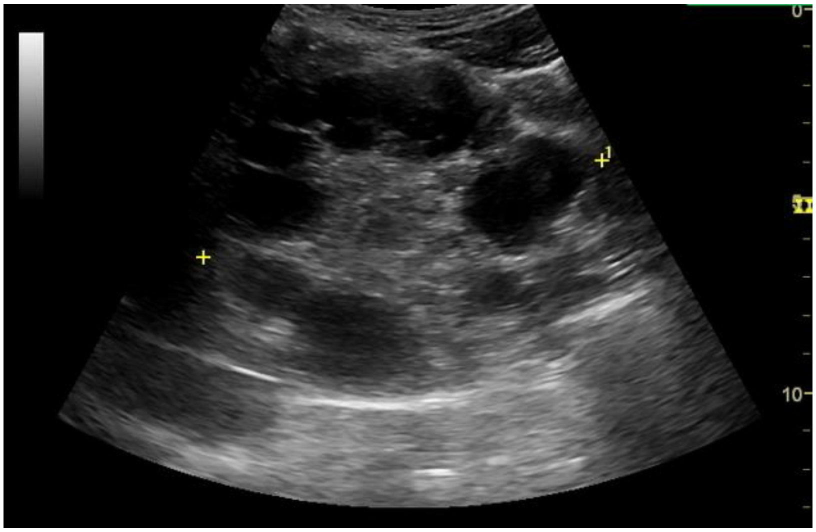

Figure 9: Well defined, multiseptated, anechoic cyst /with double wall sign/ in the liver. WHO’s classification – CE2.

Patient 10

Female, 46 age

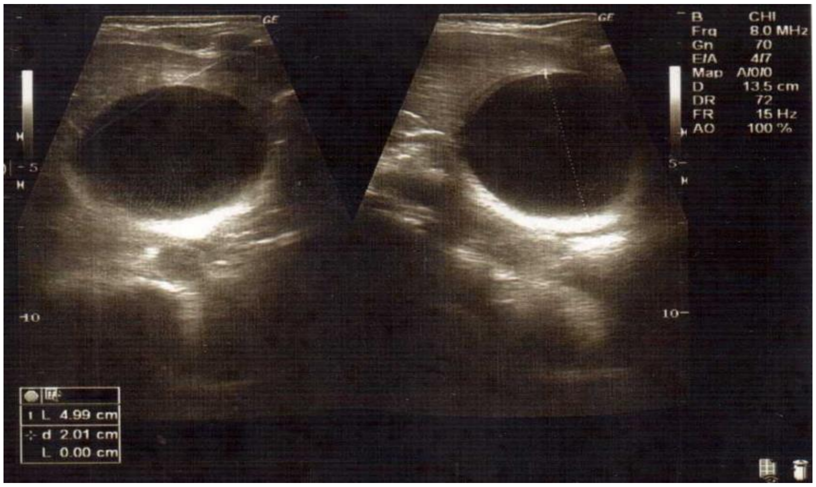

Figure 10: 4.0x3.5cm, anechoic cyst /with double wall sign/ in the liver. WHO’s classification – CE1

Patient 11

Female, 53 age

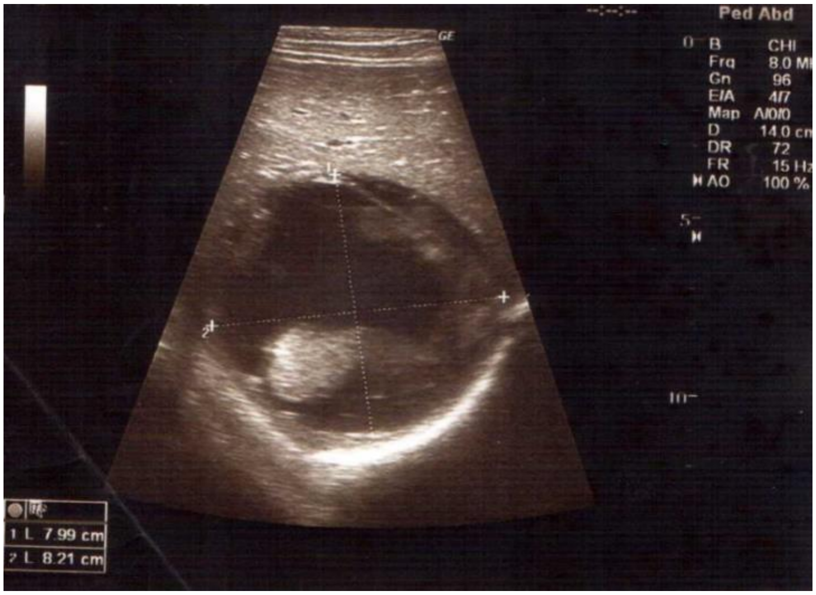

Figure 11: Anechoic, univesicular cystic with detached layers in the liver. WHO's classification – CE3.

Patient 12

Female, 55 age

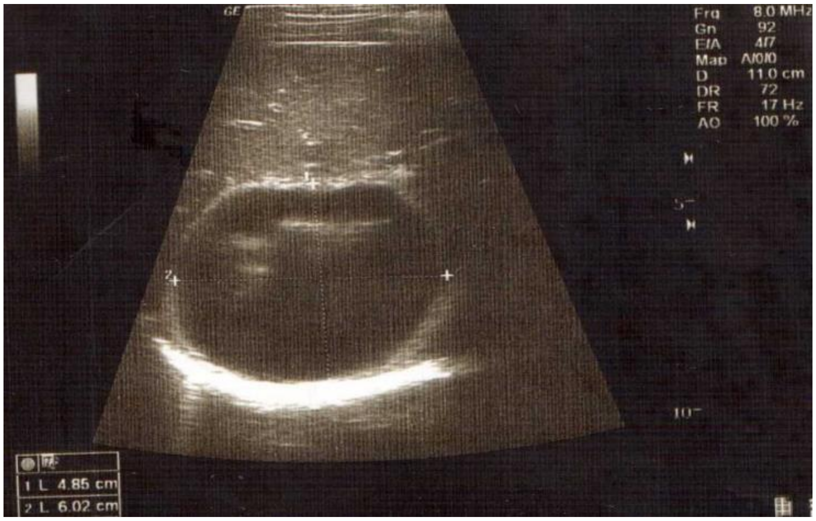

Figure 12: 4.8x6.0cm, anechoic cyst /with double wall sign/ in the liver. WHO's classification – CE1

Patient 13

Male 65 age:

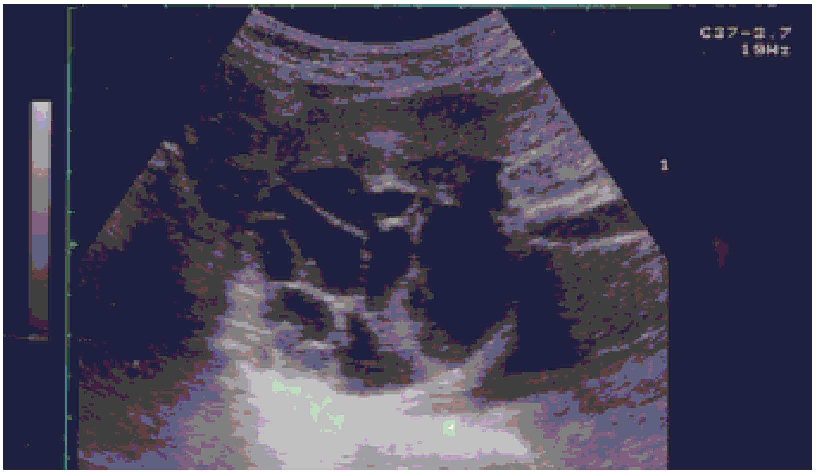

Figure 13: Well defined, multiseptated, anechoic cyst /with double wall sign/ in the liver. WHO’s classification – CE2.

ULTRASOUND FIGURES IN PATIENTS OF KHUVSGUL PROVINCE

Patient 1

Female, 12 age

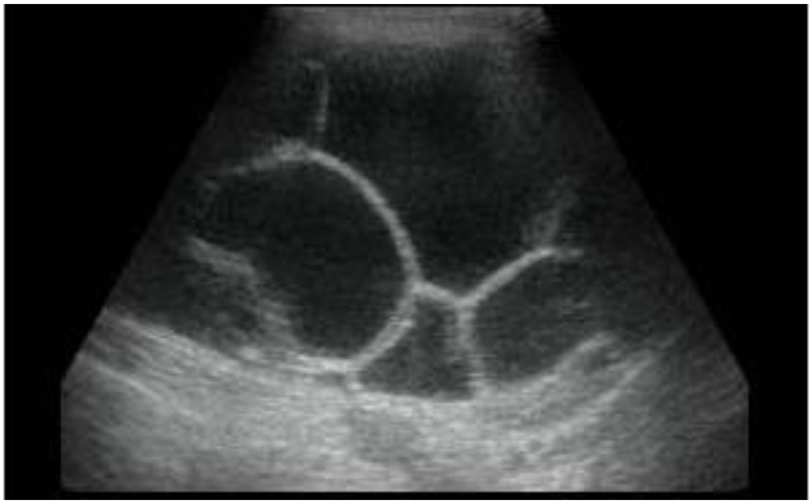

Figure 14: Well defined, multiseptated, anechoic cyst /with double wall sign/ in the liver. WHO's classification – CE2.

Patient 2

Female, 45 age

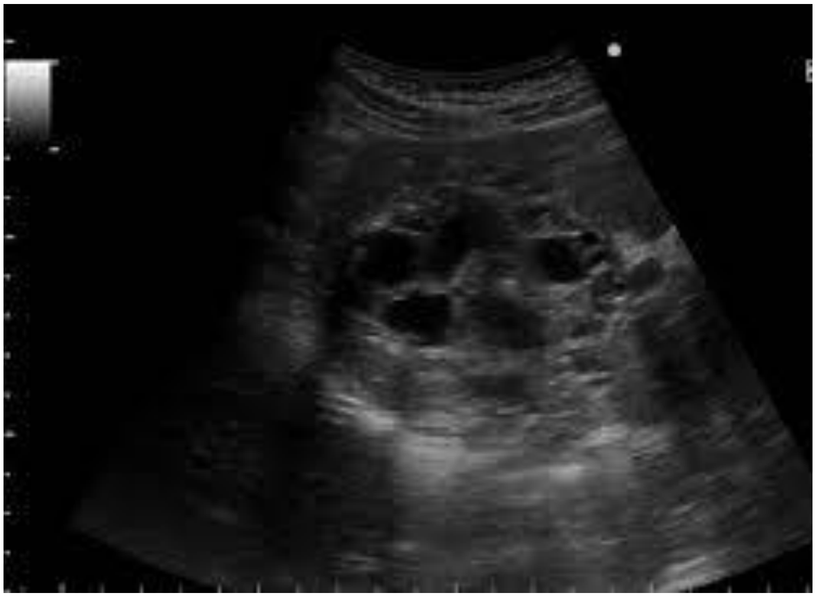

Figure 15: Well defined, multiseptated, anechoic cyst /with double wall sign/ in the liver. WHO's classification – CE2.

Patient 3

Female, 61/age

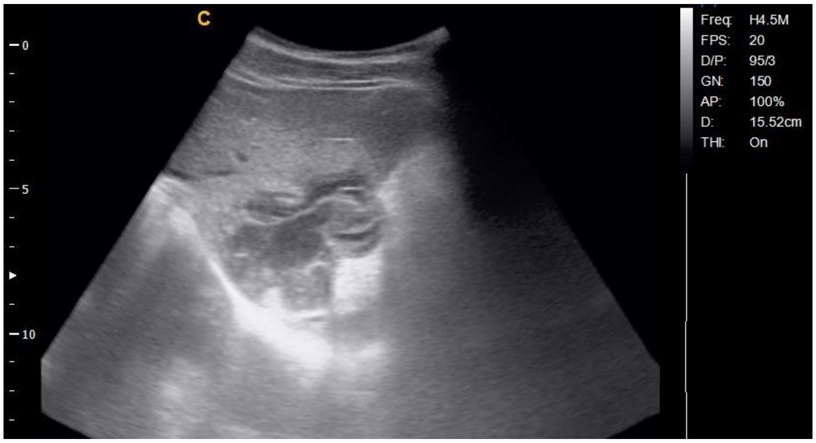

Figure 16: 5.5x4.9 cm, well defined, anechoic, univesicular cystic with detached layers in the liver. WHO's classification CE3.

Patient 4

Female, 81 age

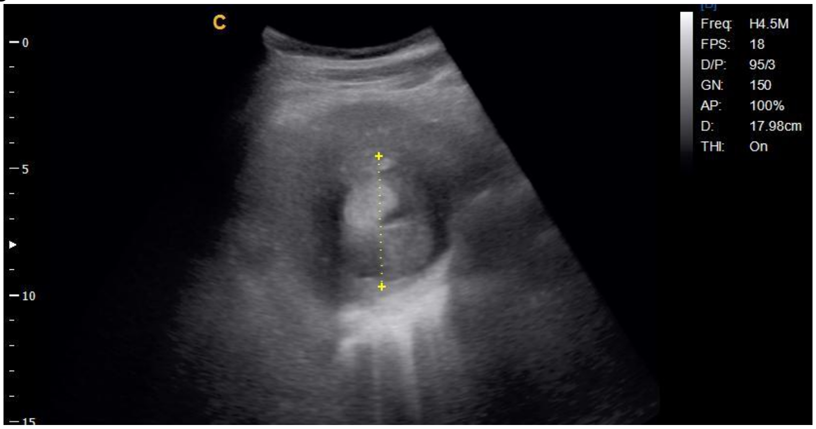

Figure 17: Heterogeneous /hyper and hypo/ echoic, cystic with double wall sign in the liver. WHO's classification – CE4.

Patient 5

Male, 57 age

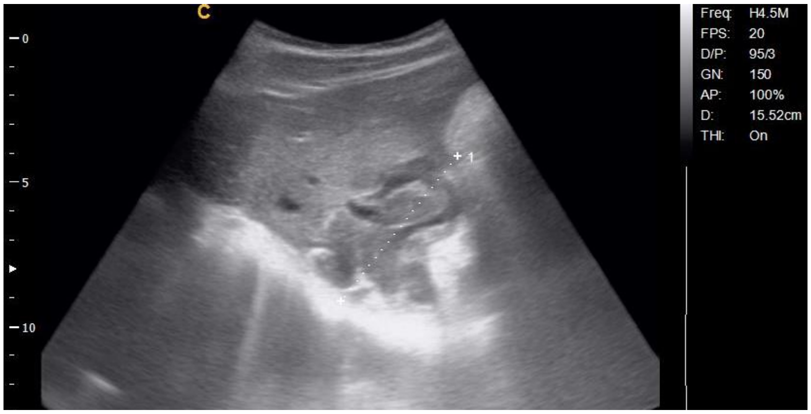

Figure 18: 5.8x6.2 cm, heterogeneous /hyper and hypo/ echoic, cystic with double wall sign in the liver

Patient 6

Female, 68 age

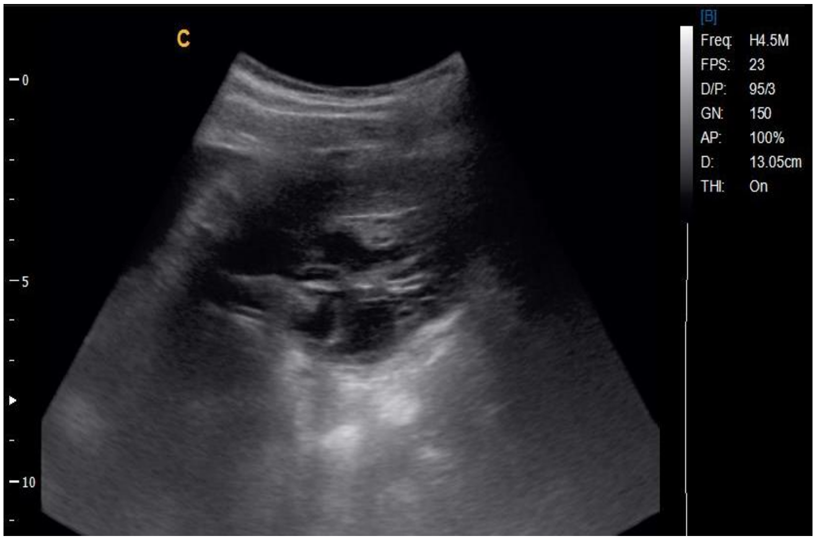

Figure 19: 5.2x4.9 cm, well defined, multiseptated, anechoic cyst /with double wall sign/ in the liver

Patient 7

Male, 51 age

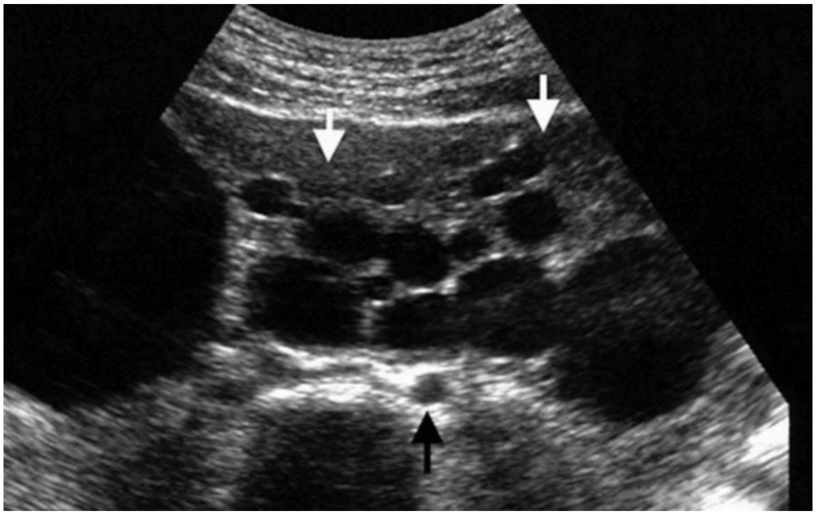

Figure 20: Well defined, multiseptated, anechoic cyst /with double wall sign/ in the liver. WHO’s classification CE2.

Patient 8

Male, 75 age

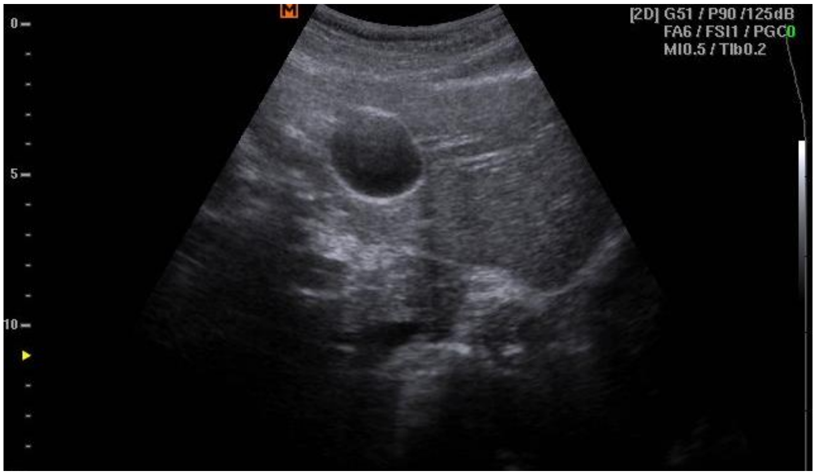

Figure 21: 4.5x4.2cm, anechoic cyst /with double wall sign/ in the VII and VI segments of the liver. WHO’s classification – CE1

ULTRASOUND FIGURES IN PATIENTS OF UMNUGOVI PROVINCE

Patient 1
Male, 16 age

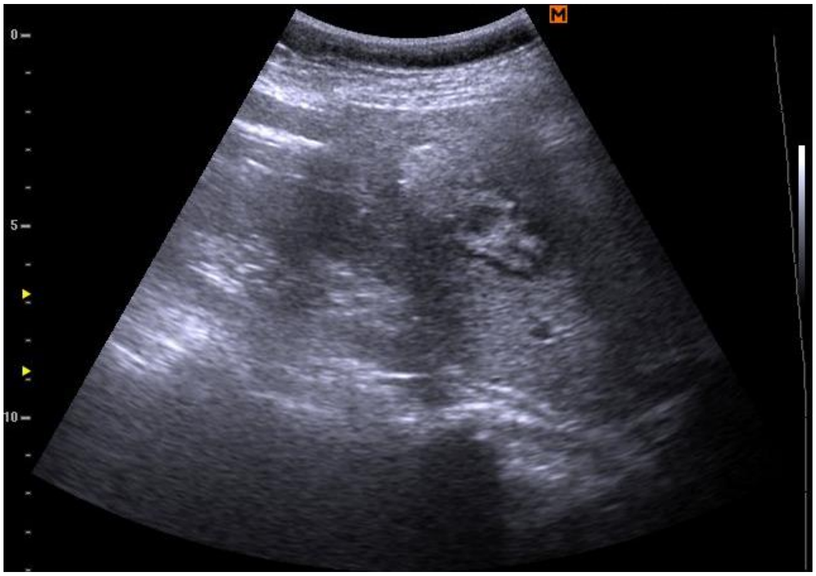

Figure 22: 3.6x2.79 cm, heterogeneous /hyper and hypo/ echoic, cystic with double wall sign in the liver. WHO's classification –CE 4.

Patient 2
Female, 45 age

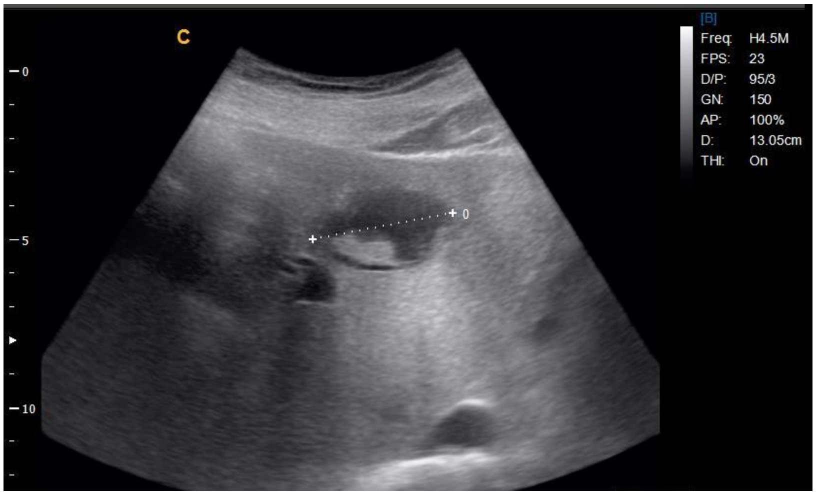

Figure 23: 3.5x2.8 cm well defined, univesicular cystic with detached layers in the 5 and 4 segment of the liver. WHO's classification – CE3a

Patient 3

Male, 47 age

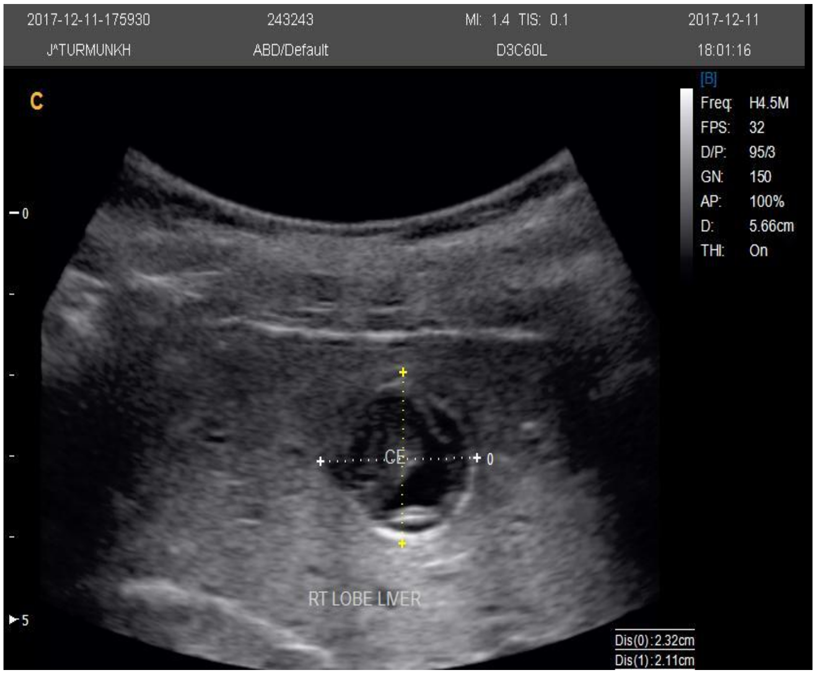

Figure 24: 2.32x2.11 cm well defined, univesicular cystic with detached layers in the 5 segment of the liver. WHO's classification – CE3a

Patient 4

Female, 6 age

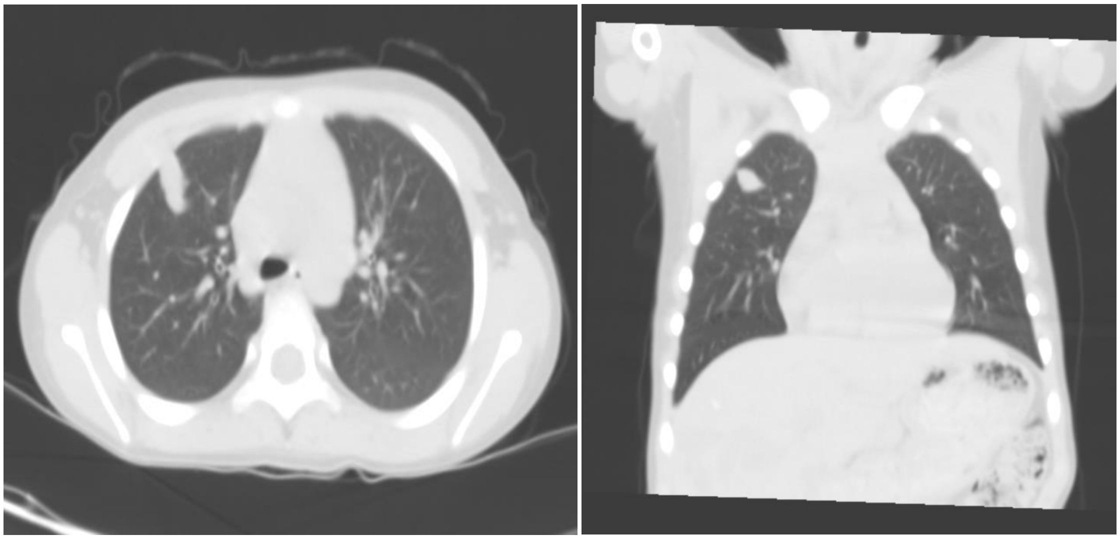

Figure 25: Chest Computed tomography without contrast (lung window) showing solitary oval shaped fluid-filled cystic lesion in right lung upper lobe parenchyma

Patient 5

Female, 71 age

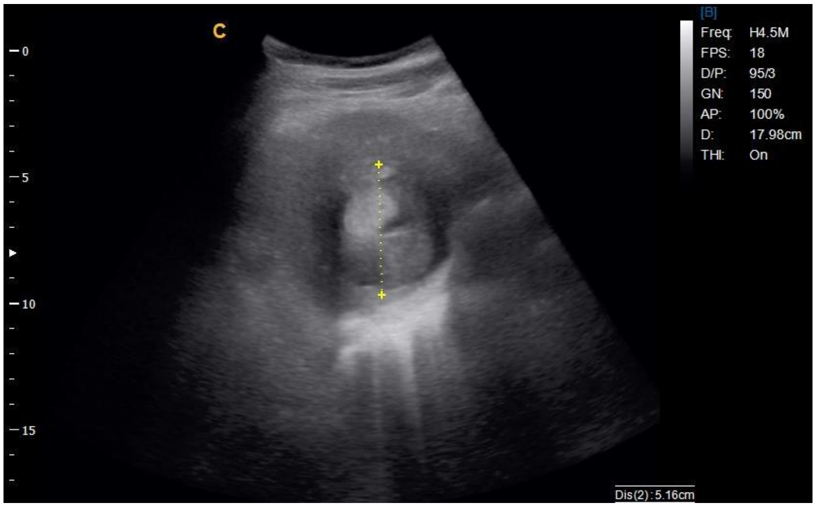

Figure 26: 6.9x5.8 cm, heterogeneous /hyper and hypo/ echoic, cystic with double wall sign in the liver. WHO’s classification – CE4.

Patient 6

Female, 40 age

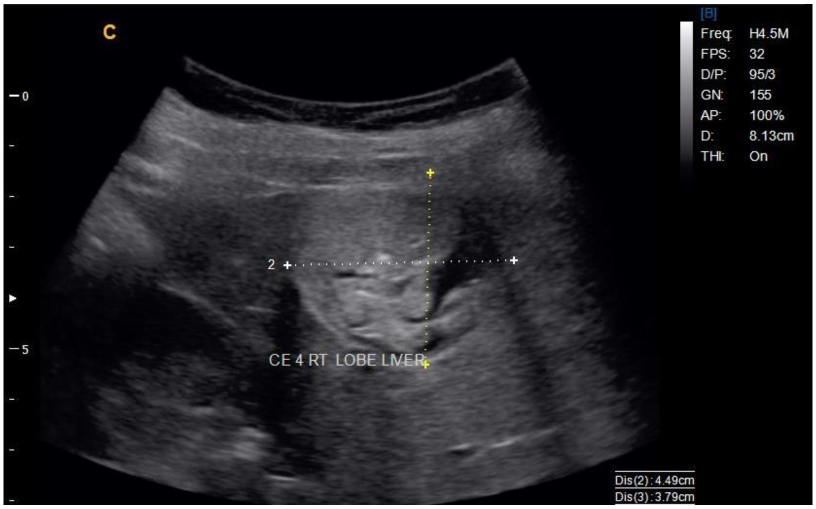

Figure 27: 4.49x3.79 cm, heterogeneous /hyper and hypo/ echoic, cystic with double wall sign in the V and VI segment of the liver. WHO’s classification – CE4.

Patient 7

Female, 90 age

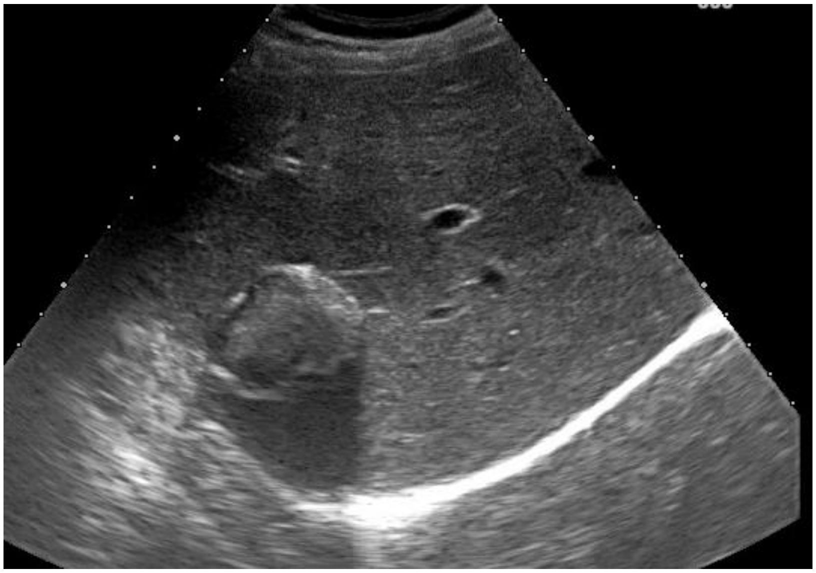

Figure 28: 2.5 cm, calcified wall with acoustic shadow, solid cyst in the 8 segment of liver WHO's classification CE5

Patient 8

Male, 16 age

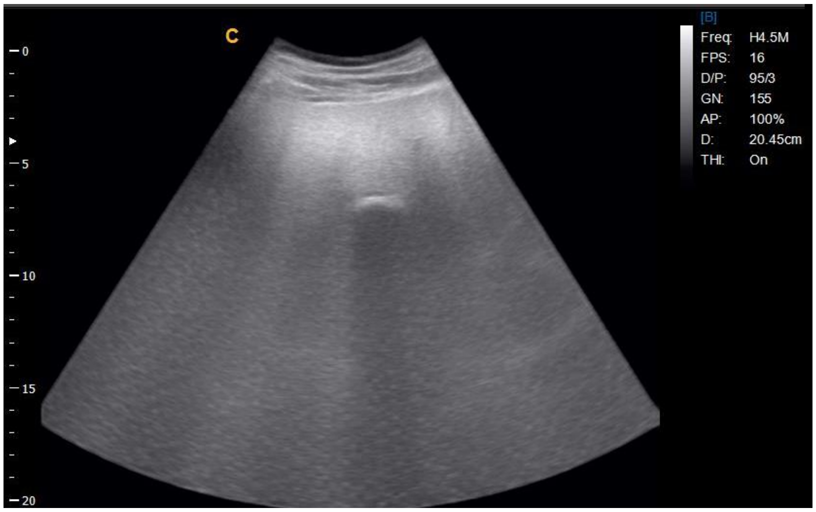

Figure 29: 3.3 cm, calcified wall with acoustic shadow, solid cyst in the liver WHO's classification CE5

Patient 9

Male, 13 age

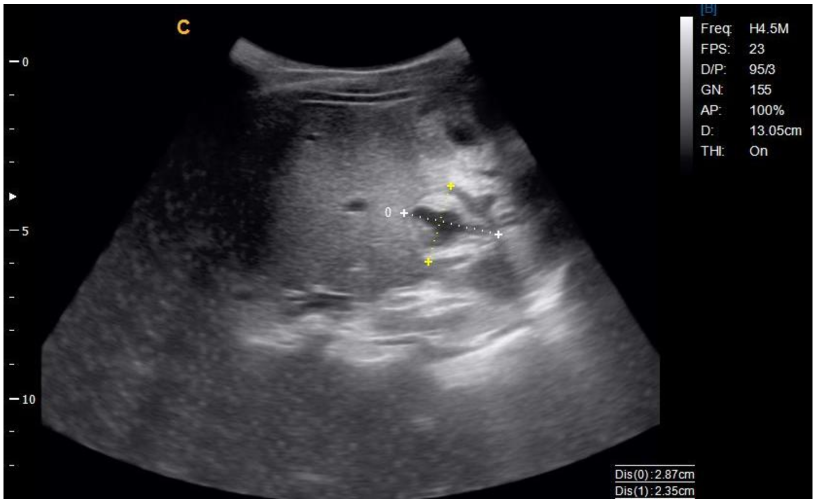

Figure 30: 2.87x2.35 cm, heterogeneous /hyper and hypo/ echoic,

cystic with double wall sign in the VII segment of the liver. WHO’s classification – CE4.

Patient 10

Male, 62 age

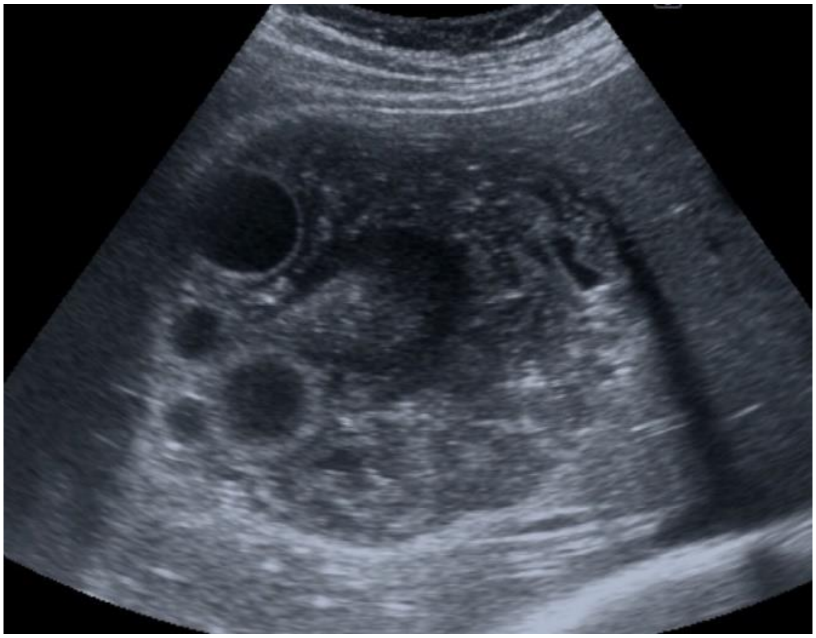

Figure 31: 3.2x3.35 cm, heterogeneous /hyper and hypo/ echoic,

cystic with double wall sign in the liver. WHO’s classification – CE4.

Patient 11

Male, 35 age

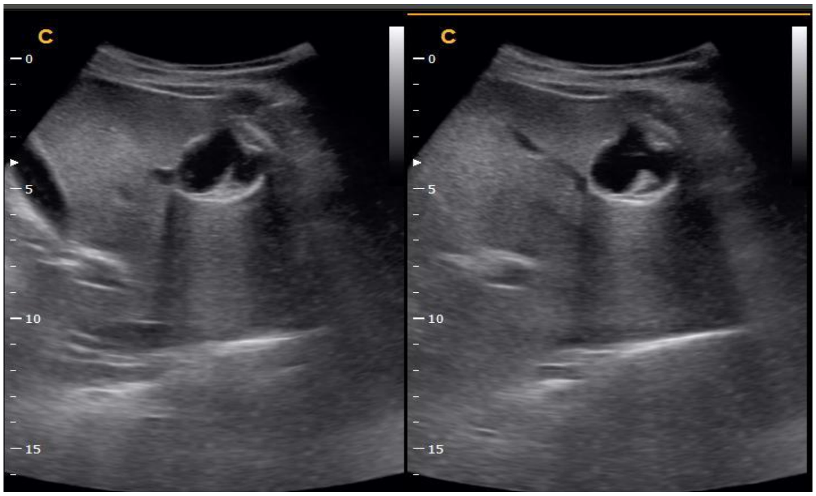

Figure 32: Well defined, anechoic, univesicular cystic with detached layers in the IV segment of the liver. WHO’s classification –CE3a

Patient 12

Male, 70 age

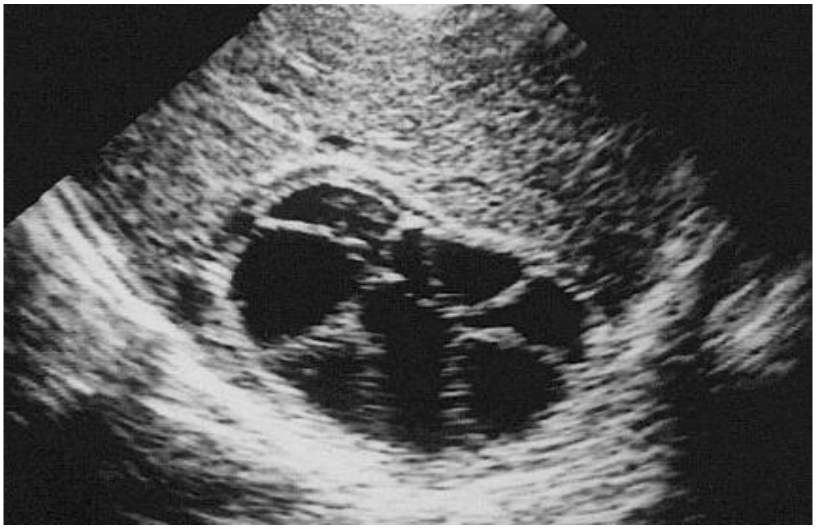

Figure 33: 3.5x3.7cm, well defined, multiseptated, anechoic cyst /with double wall sign/ in the right lobe of the liver. WHO’s classification –CE2

Patient 13
Female, 54 age

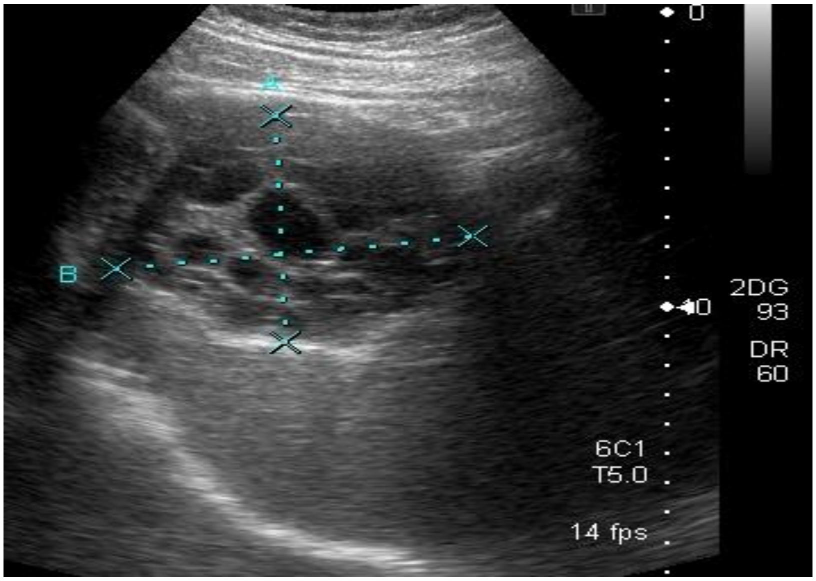

Figure 34: 4.3x4.8cm, well defined, multiseptated, anechoic cyst /with double wall sign/ in the right lobe of the liver. WHO’s classification – CE2

Patient 14
Female, 54 age

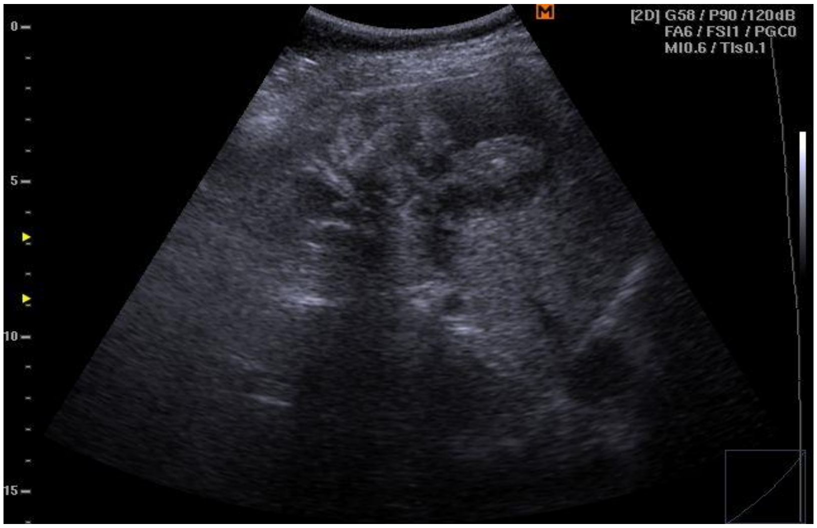

Figure 35: Heterogeneous /hyper and hypo/ echoic mass /with acoustic shadow/ in the IV segment of the liver. WHO’s classification – CE4

Patient 15

Male, 77 age

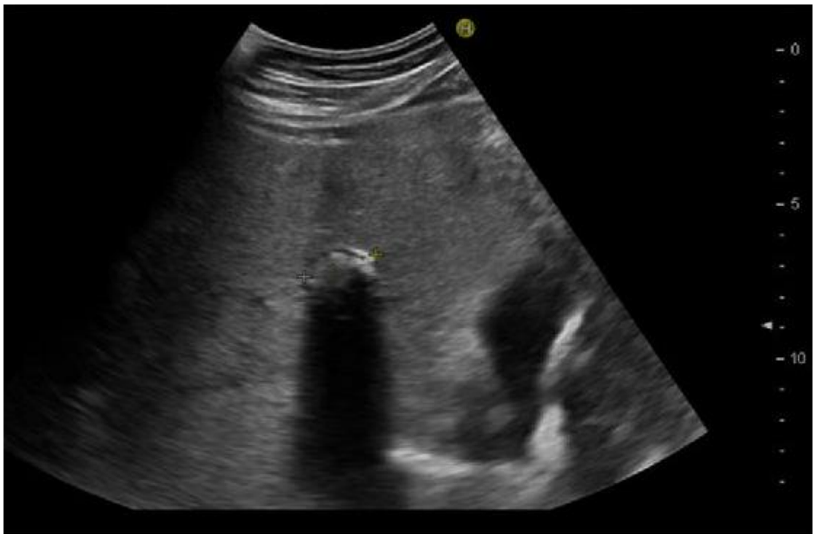

Figure 36: Calcified wall with acoustic shadow, solid cyst in the liver WHO’s classification CE5

Patient 16

Male, 8 age:

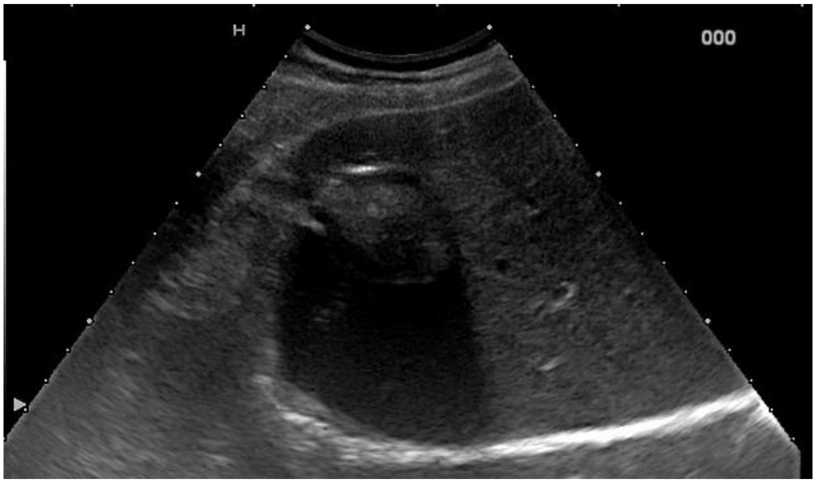

Figure 37: 3.5x4.2cm, calcified wall with acoustic shadow and solid cyst in the liver WHO’s classification CE5

Patient 17

Female, 7 age

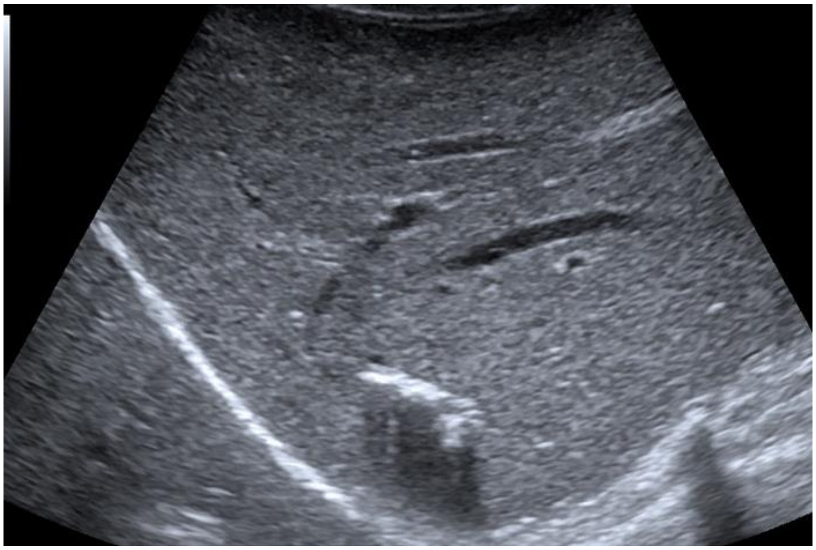

Figure 38: Calcified wall with acoustic shadow, solid cyst in the liver WHO’s classification CE5

Patient 18

Female, 29 age

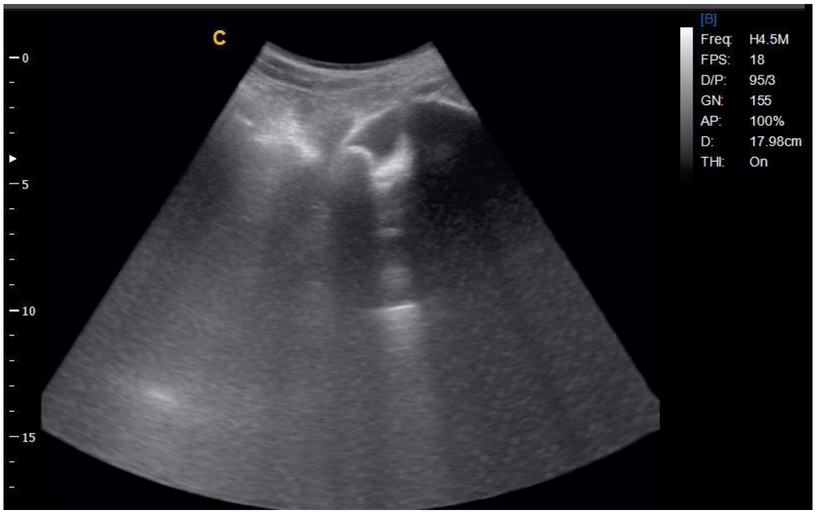

Figure 39: 6.3x5.0cm, calcified wall with acoustic shadow and solid cyst in the liver WHO’s classification CE5

Patient 19

Female, 36 age

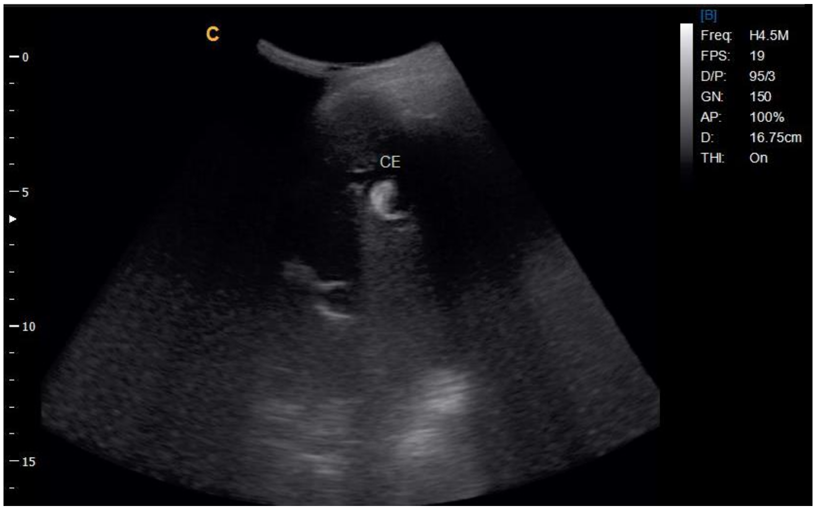

Figure 40: 1.5x1.7cm, calcified wall with acoustic shadow and solid cyst in the liver WHO's classification CE5

Patient 20

Female, 24 age

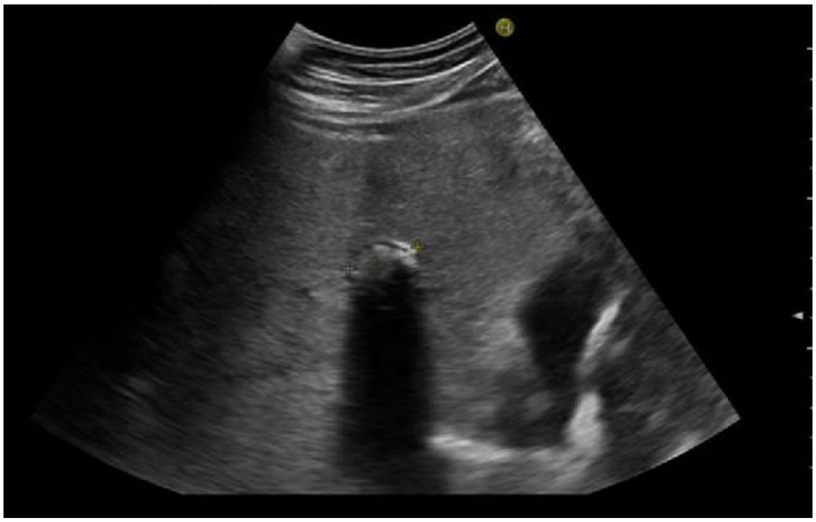

Figure 41: Calcified wall with acoustic shadow, solid cyst in the liver WHO's classification CE5

Patient 21

Male, 9 age

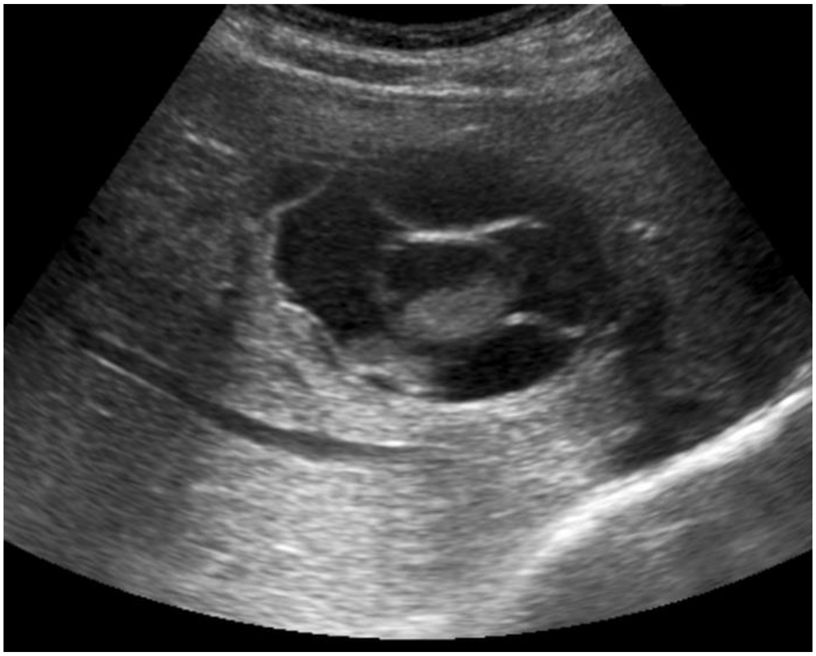

Figure 42: 3.8x3.5 cm, well defined, anechoic, univesicular cystic
with detached layers in the IV segment of the liver. WHO's classification – CE3a

Patient 22

Female, 32 age

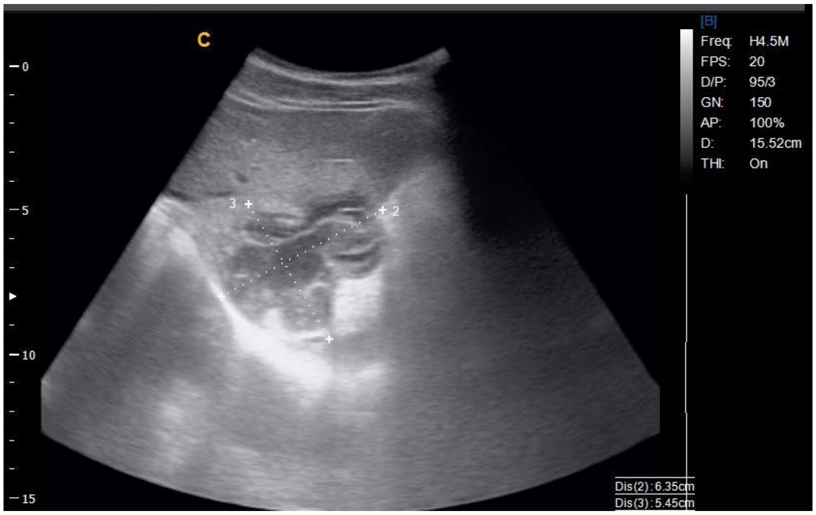

Figure 43: 6.35x5.45 cm, well defined, anechoic, univesicular cystic

with detached layers in the left lobe of the liver. WHO's classification – CE3a

Patient 23

Male, 79 age

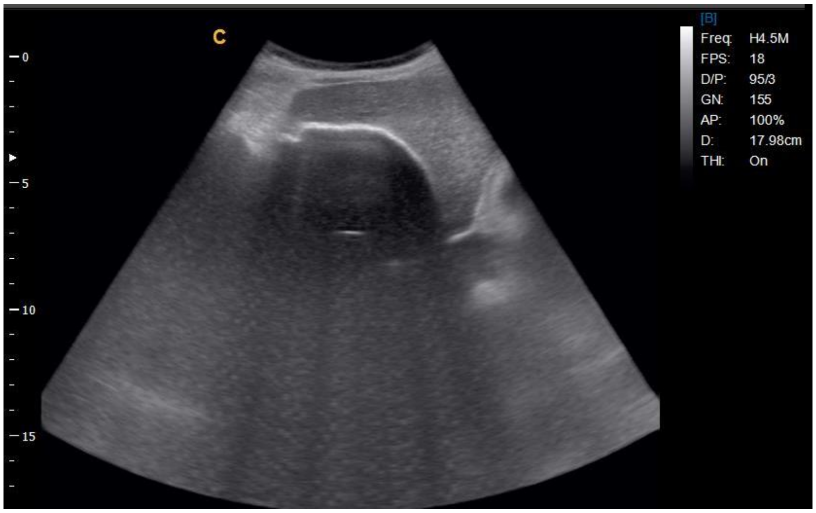

Figure 44: 6.8x7.9 cm, calcified wall with acoustic shadow and solid cyst in the liver WHO’s classification CE5

Patient 24

Female, 57 age

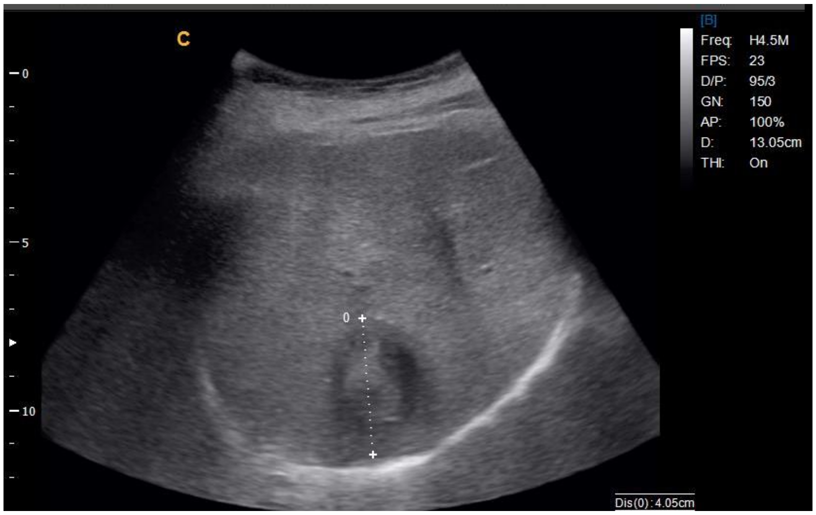

Figure 45: D=4.05 cm, heterogeneous /hyper and hypo/ echoic, cystic with double wall sign in the right lobe of liver. WHO’s classification – CE4.

Patient 25

Female, 45 age

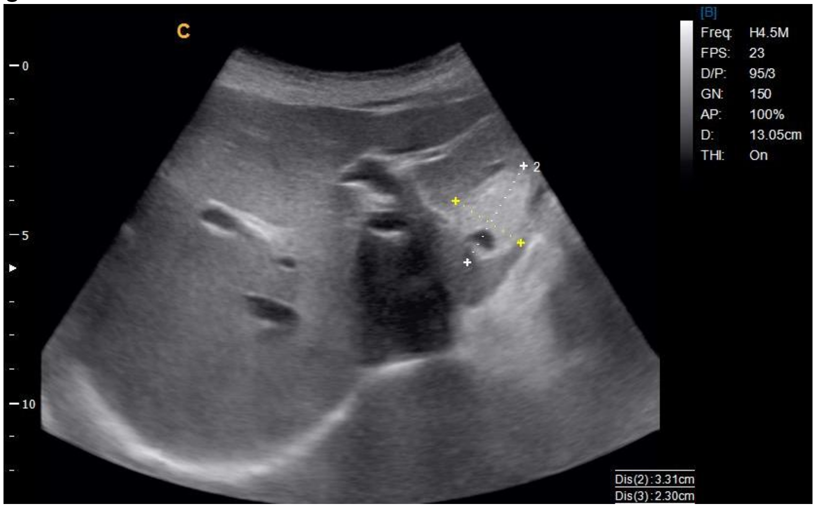

Figure 46: 3.31x2.3 cm, cyst with daughter vesicles in solid matrix and double wall sign in the II segment of liver. WHO's classification – CE3b

Patient 26

Female, 46 age

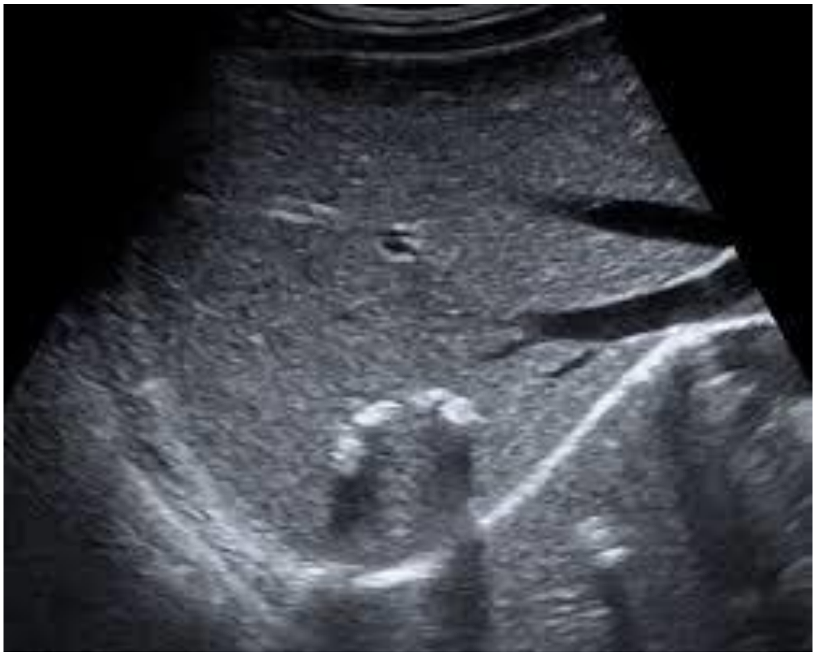

Figure 47: 2.2x2.4cm, calcified wall with acoustic shadow and solid cyst in the liver WHO's classification CE5

Patient 27

Female, 41 age

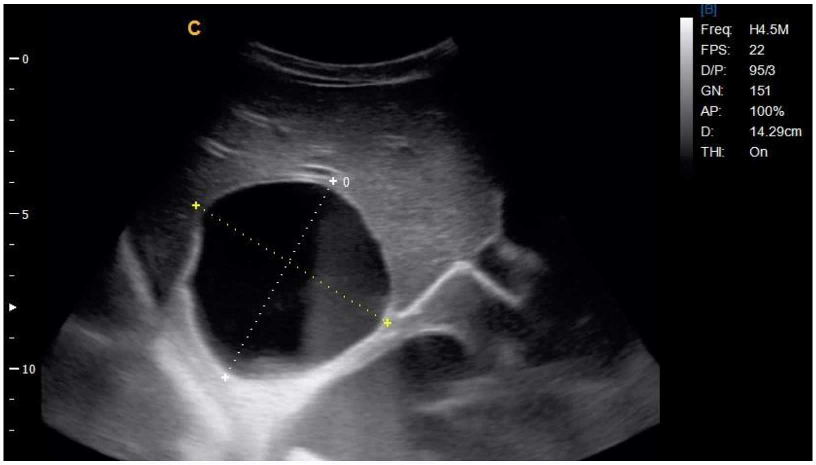

Figure 48: 5.6x5.7 cm, anechoic cyst /with double wall sign/ in the VII and VIII segments of the liver. WHO's classification – CE1

Patient 28

Female, 74 age

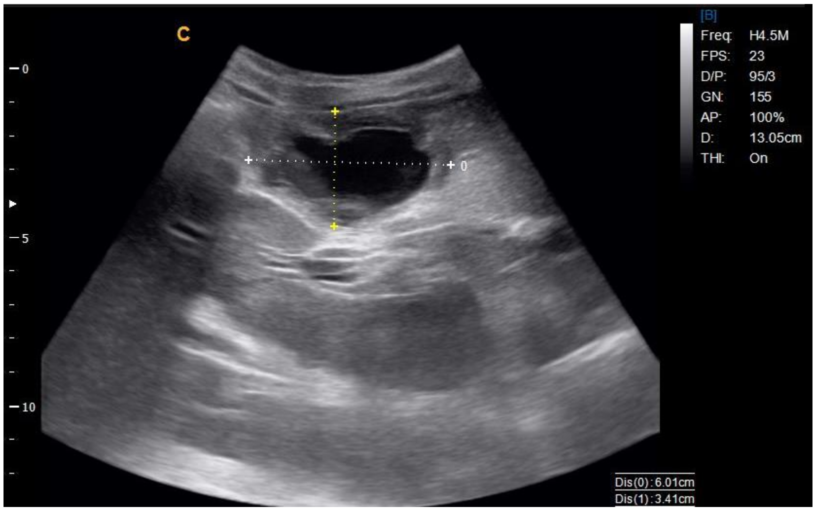

Figure 49: 6.01x3.41 cm, well defined, anechoic, univesicular cystic with detached layers in the right lobe of the liver. WHO's classification – CE3a.

Patient 29

Female, 47 age

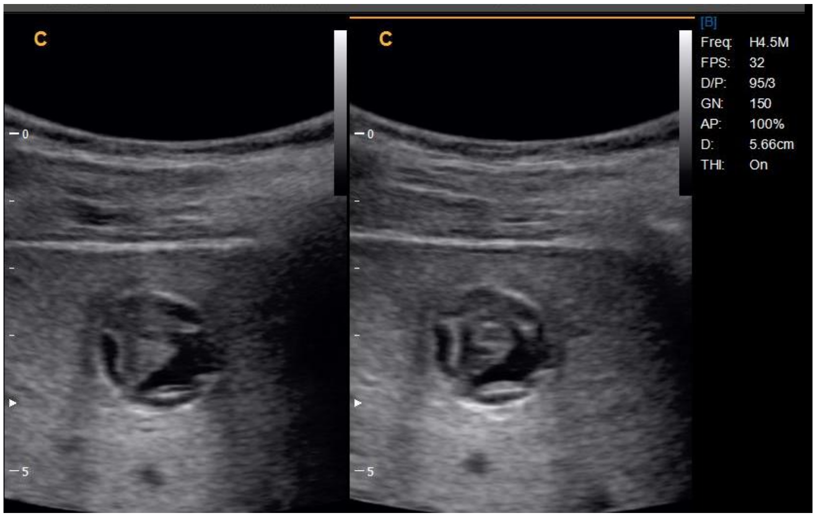

Figure 50: 1.7x1.5 cm, well defined, anechoic, univesicular cystic with detached layers in the right lobe of the liver. WHO's classification – CE3a.

Patient 30

Female, 34

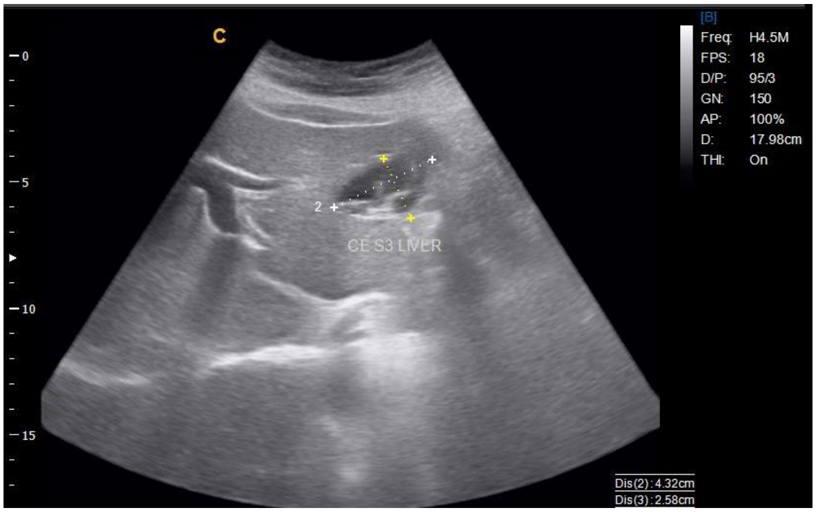

Figure 51: 4.32x2.52 cm, well defined, anechoic, univesicular cystic with detached layers in the liver. WHO's classification – CE3a.

Patient 31

Male, 51 age

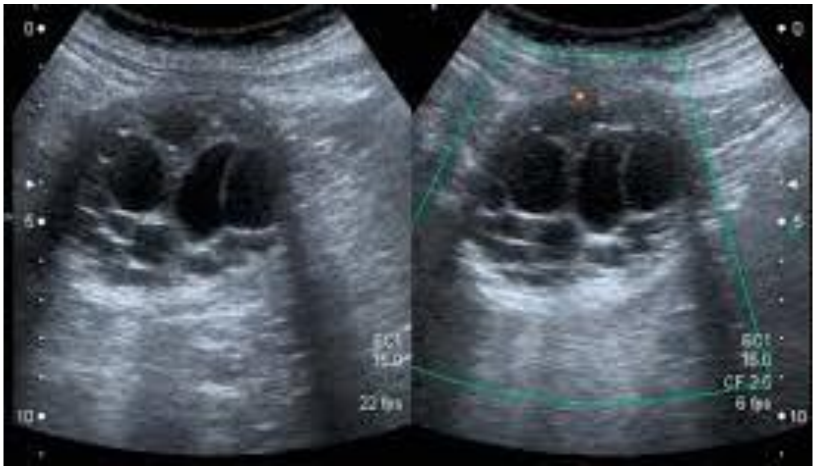

Figure 52: Well defined, multiseptated, anechoic cyst /with double wall sign/ in the right lobe of the liver. WHO’s classification – CE2

Patient 32

Female, 72 age

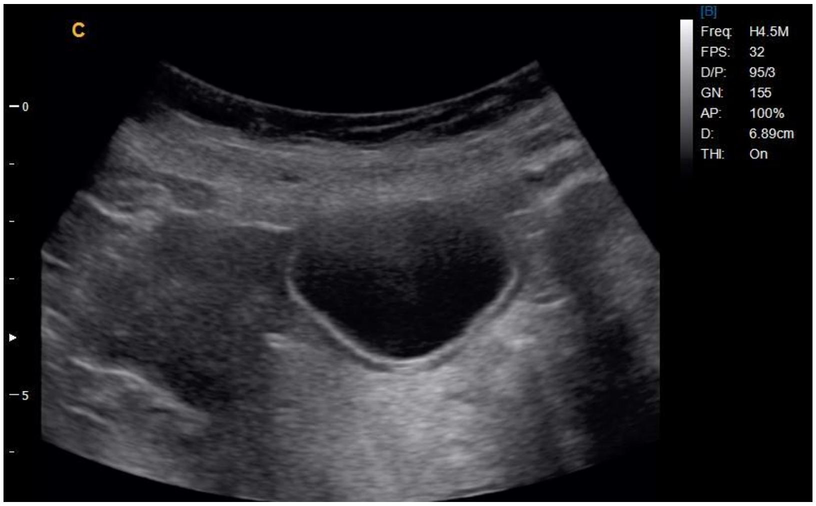

Figure 53: 2.1x1.9 cm, well defined, anechoic cyst /with double wall sign/ in the liver. WHO’s classification – CE1

Patient 33

Female, 76 age

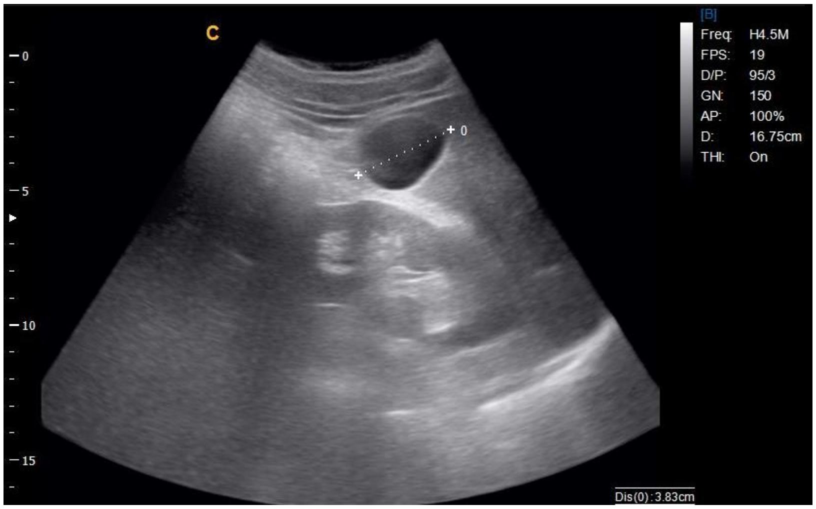

Figure 54: D=3.83 cm, well defined, anechoic cyst /with double wall/ sign in the VI segment of the liver. WHO’s classification – CE1

Patient 34

Female, 73 age

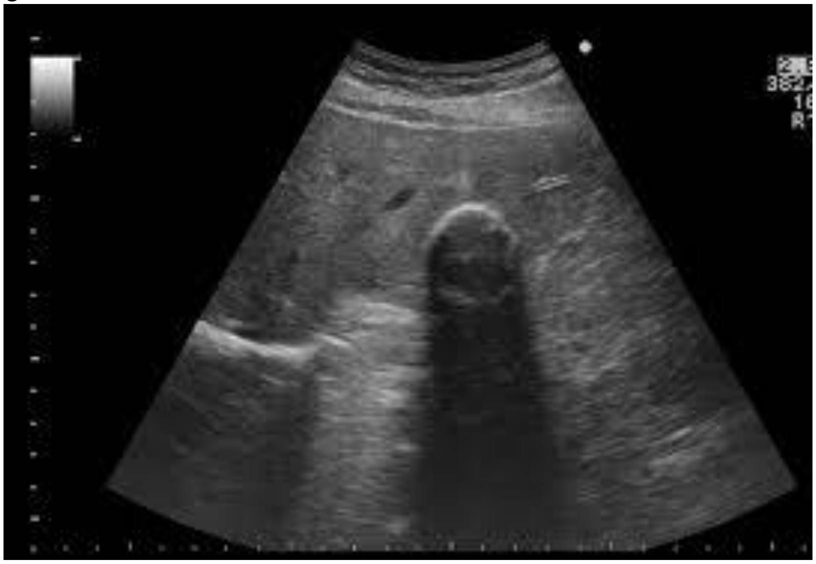

Figure 55: 2.8x3.0cm, calcified wall with acoustic shadow and solid cyst in the liver WHO’s classification CE5

ULTRASOUND FIGURES IN PATIENTS OF KHOVD PROVINCE

Patient 1
Female, 61 age

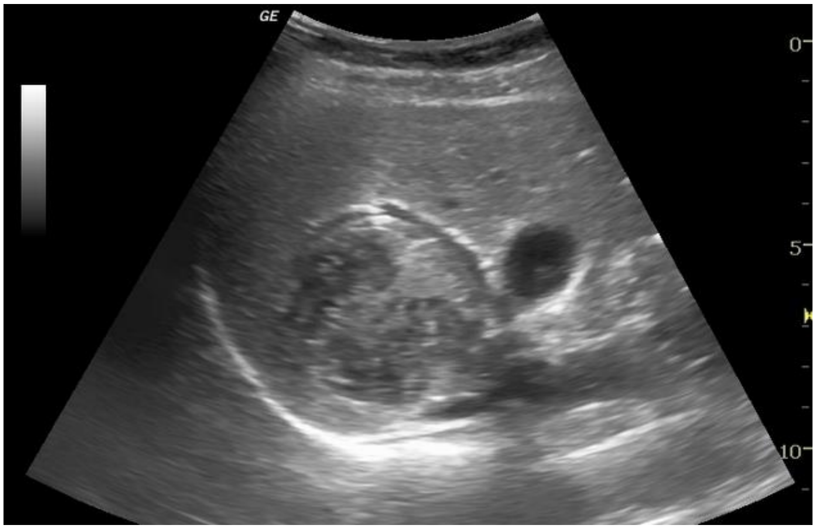

Figure 56: Heterogeneous /hyper and hypo/ echoic mass /with acoustic shadow/ in the liver. WHO’s classification – CE4.

Patient 2
Female, 85 age

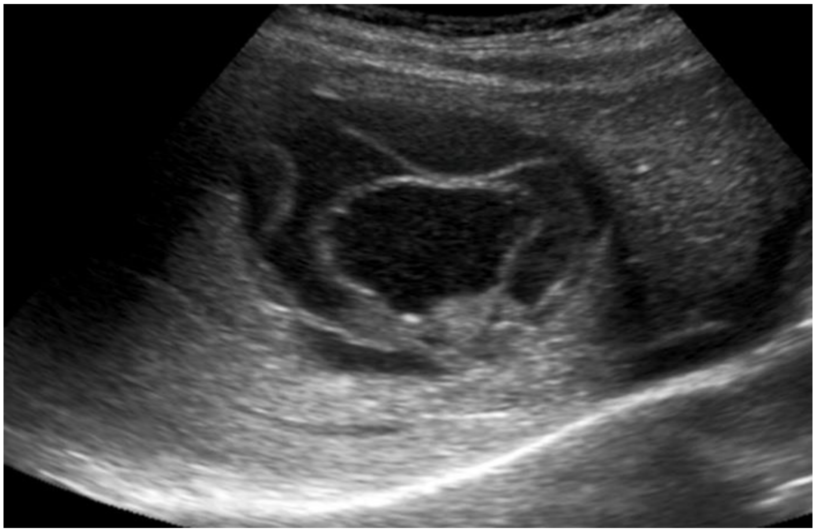

Figure 57: Well defined, anechoic, univesicular cystic with detached layers in the liver. WHO’s classification – CE3.

Patient 3
Male, 56 age

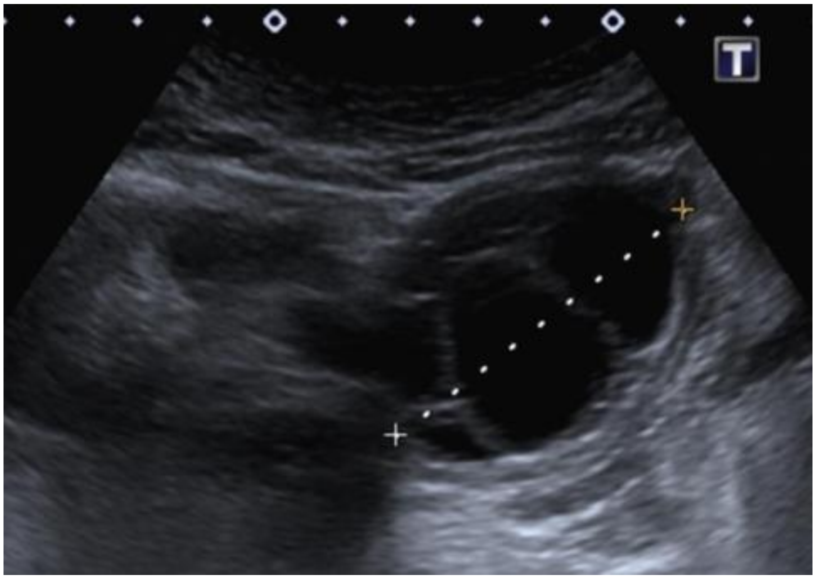

Figure 58: 3.6x3.4 cm, well defined, multiseptated, anechoic cyst /with double wall sign/ in the liver. WHO’s classification – CE2

Patient 4
Female, 53 age

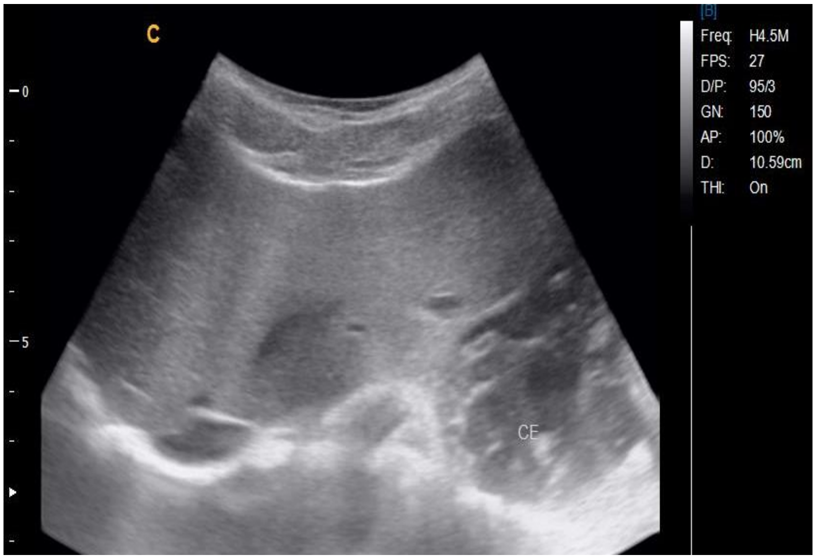

Figure 59: 4.2x4.8 cm, well defined, anechoic, univesicular cystic with detached layers in the liver. WHO’s classification – CE3

Patient 5

Male, 54 age

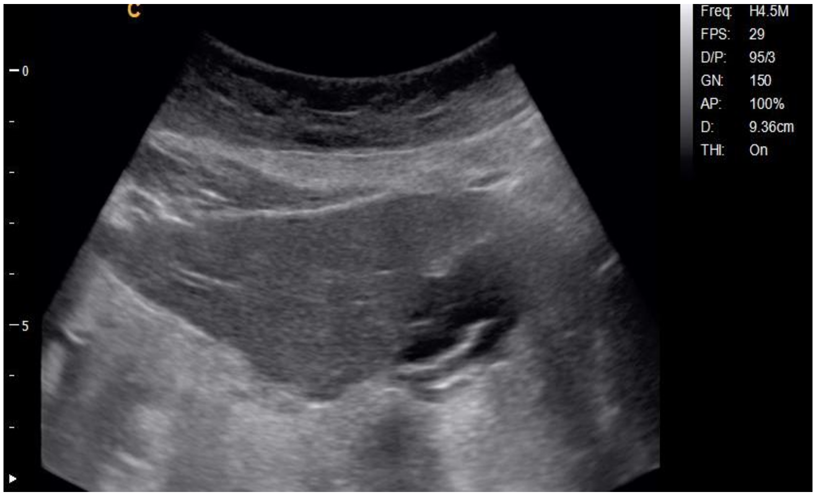

Figure 60: 2.9x3.5 cm, well defined, multiseptated,

anechoic cyst /with double wall sign/ in the liver. WHO’s classification – CE2

Patient 6

Female, 62 age

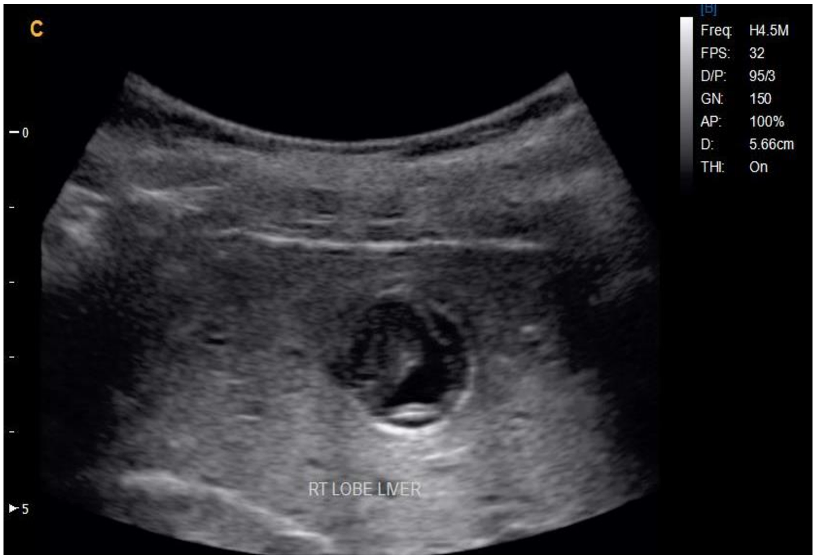

Figure 61: 3.2x2.9 cm, well defined, anechoic, univesicular cystic with

detached layers in the right lobe of liver. WHO’s classification – CE3

Patient 7
Male, 54 age

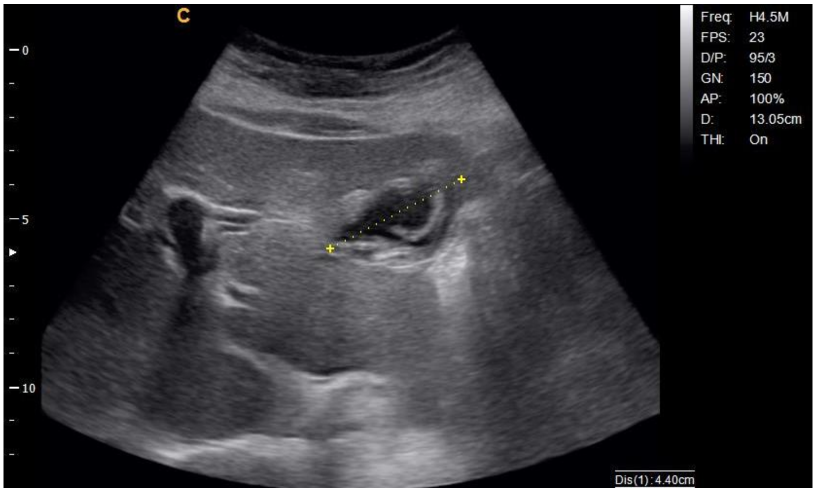

Figure 62: 3.6x4.2cm, well defined, anechoic, univesicular cystic with detached layers in the liver. WHO’s classification – CE3

Patient 8
Male, 14 age

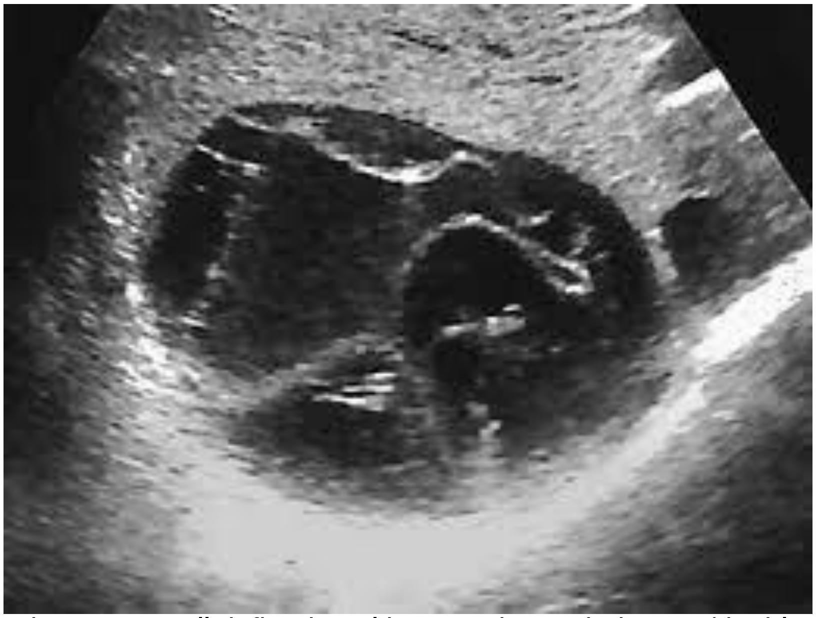
Figure 63: Well defined, multiseptated, anechoic cyst /double wall sign/ in the liver. WHO’s classification – CE2.

Patient 9
Female, 12 age

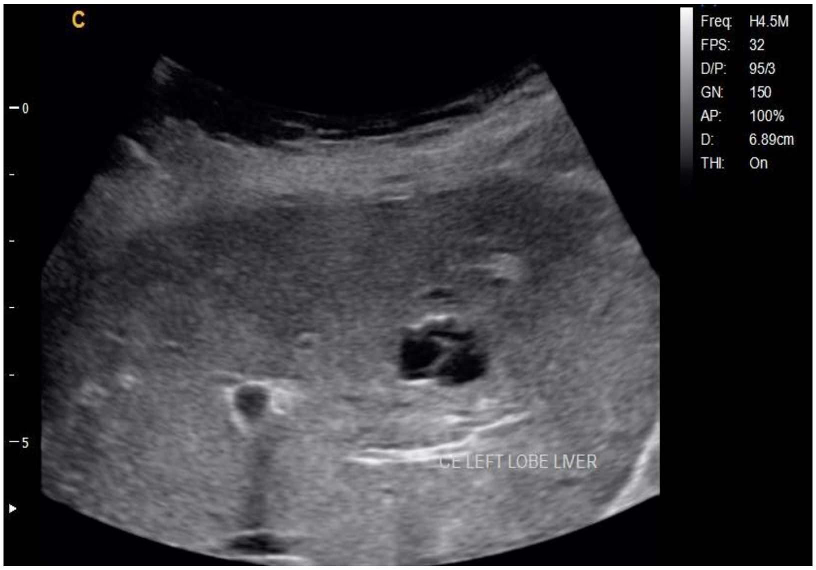

Figure 64: Well defined, multiseptated, anechoic cyst /with double wall sign/ in the left lobe of liver. WHO's classification – CE2

Patient 10
Male, 76 age

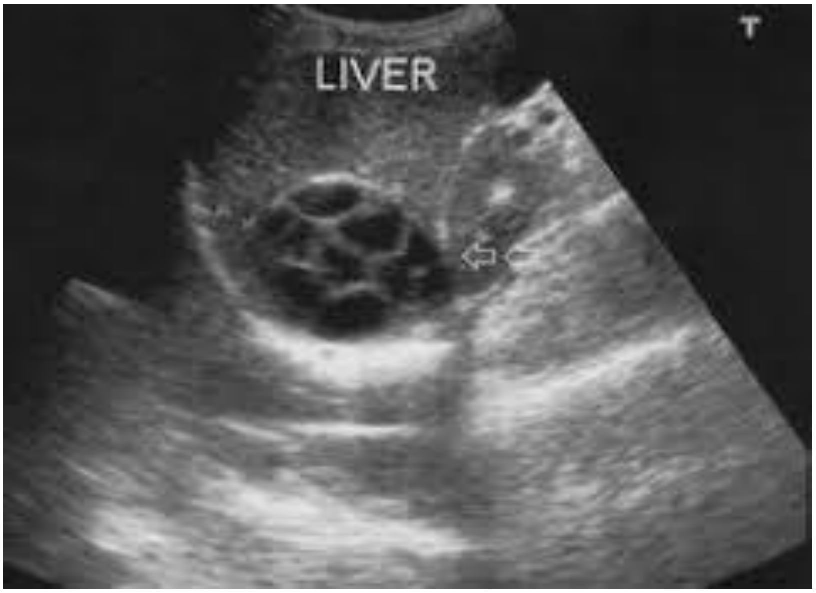

Figure 65: Well defined, multiseptated, anechoic cyst /with double wall sign/ in the liver. WHO's classification – CE2.

Patient 11

Male, 46 age

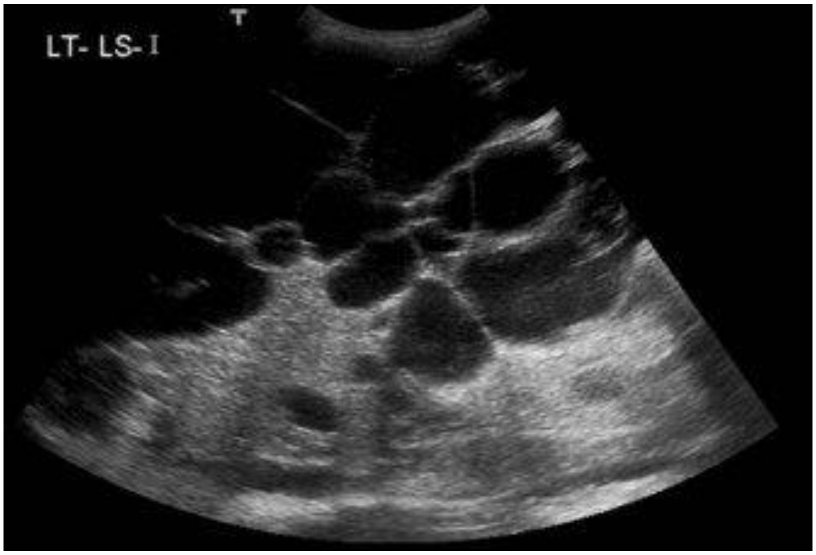

Figure 66: 4.5x5.2 cm, well defined, multiseptated, anechoic cyst /with double wall sign/ in the liver. WHO’s classification – CE2.

Patient 12

Female, 82 age

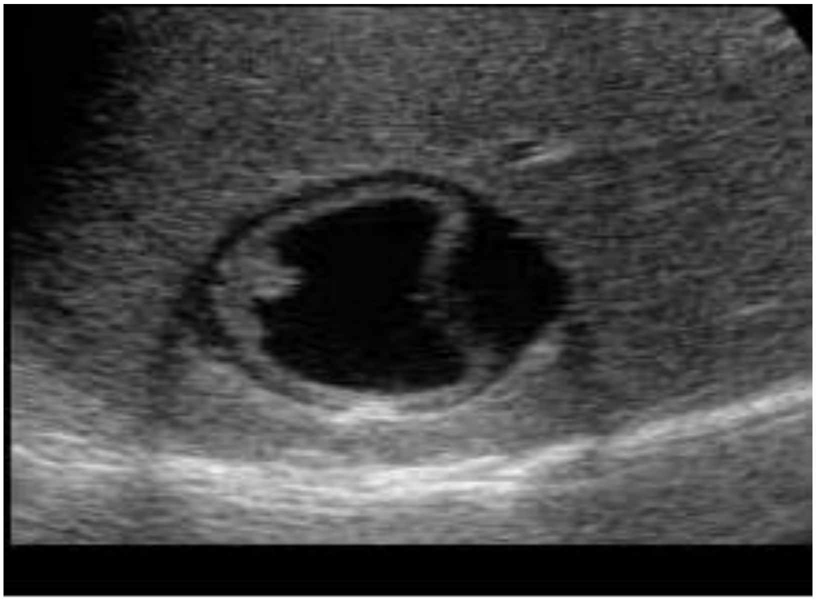

Figure 67: Well defined, anechoic, univesicular cystic with detached layers in the liver. WHO’s classification – CE3.

Patient 13
Female, 37 age

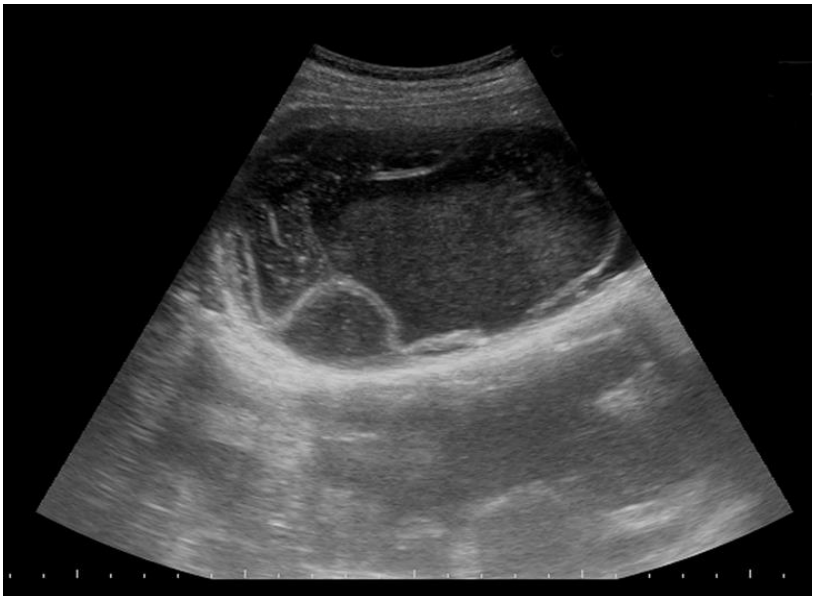

Figure 68: Well defined, anechoic, univesicular cyst

with detached layers in the liver. WHO’s classification – CE3.

Patient 14
Female, 77 age

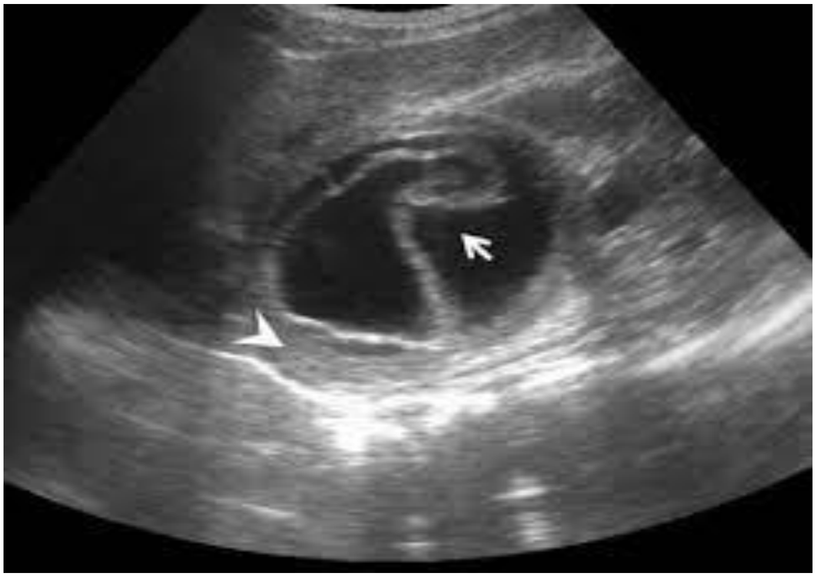

Figure 69: Well defined, anechoic, univesicular cyst

with detached layers in the liver. WHO’s classification – CE3

Patient 15

Male, 63 age

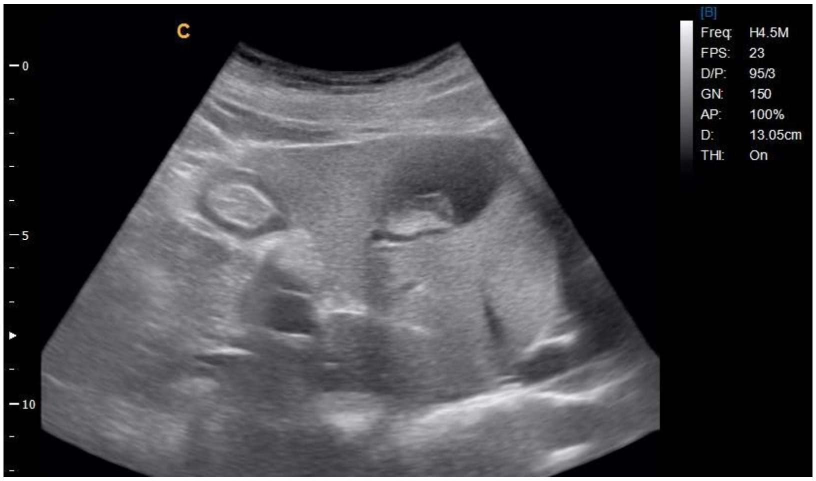

Figure 70: 3.2x3.5 cm, anechoic, univesicular cystic with detached layers in the liver. WHO’s classification – CE3.

Patient 16

Male, 64 age

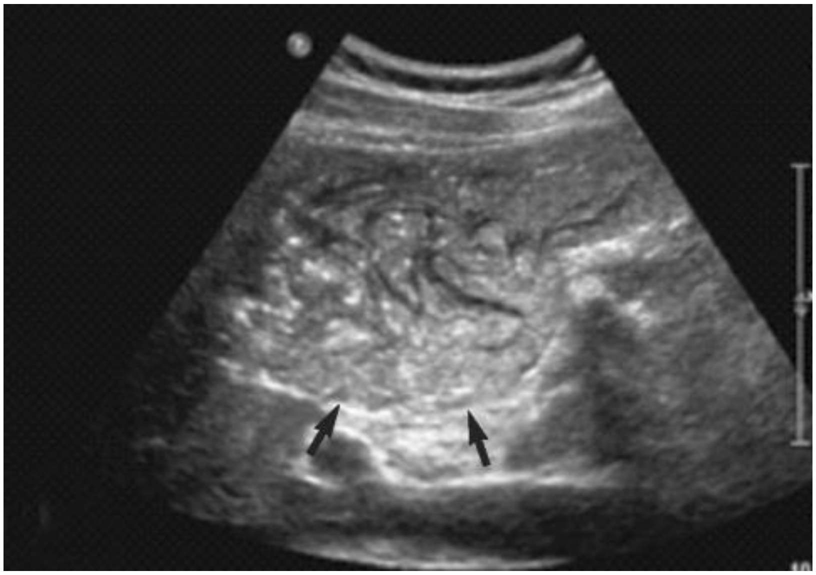

Figure 71: Heterogeneous /hyper and hypo/ echoic mass /with acoustic shadow/ in the liver. WHO’s classification – CE4.

Patient 17

Female, 77 age

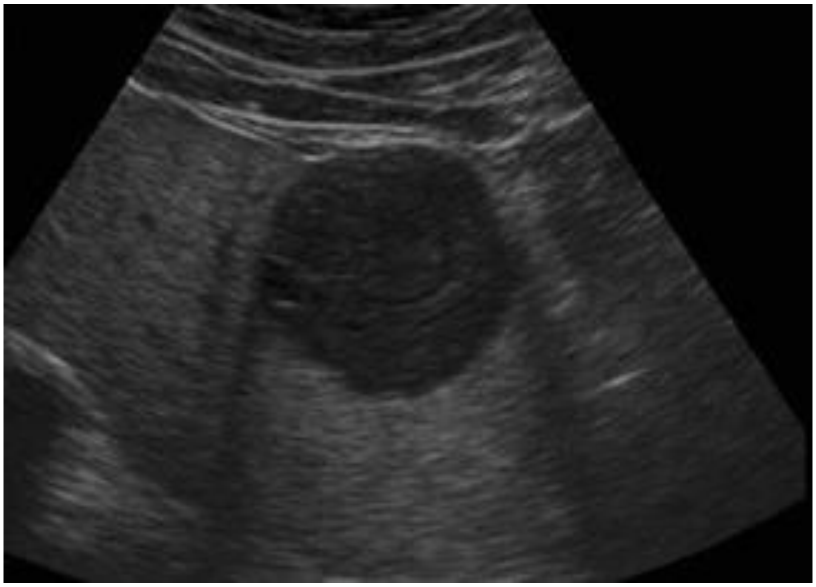

Figure 72: Heterogeneous /hyper and hypo/ echoic mass /with acoustic shadow/ in the liver. WHO's classification – CE4

Patient 18

Female, 65 age

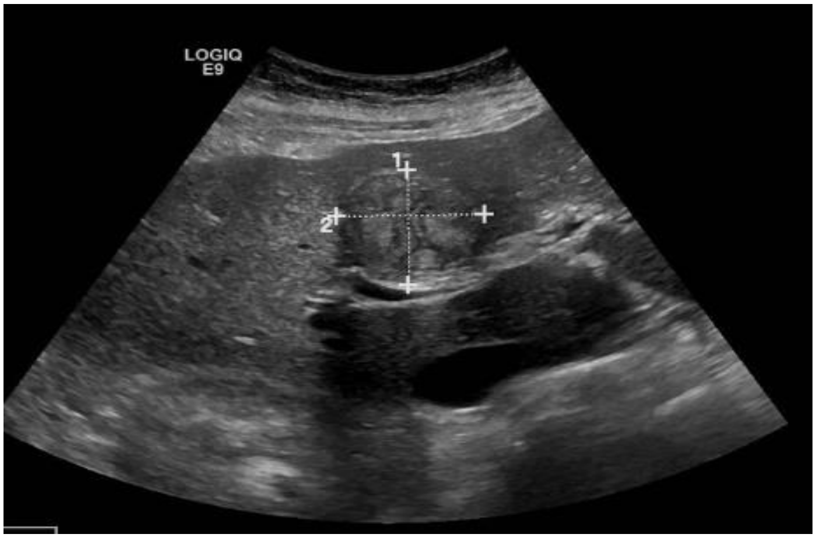

Figure 73: Heterogeneous /hyper and hypo/ echoic mass /with acoustic shadow/ in the liver. WHO's classification – CE4

Patient 19

Male, 65 age

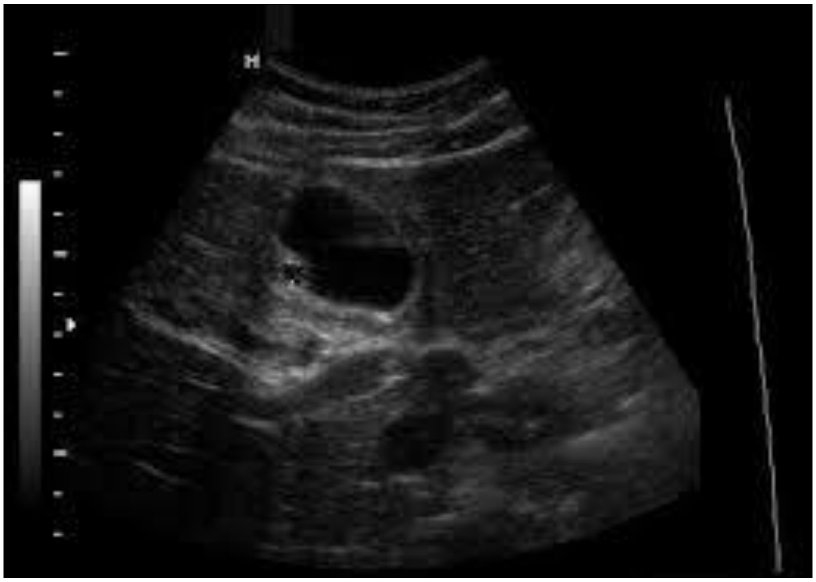

Figure 74: Well defined, anechoic, univesicular cyst with detached layers in the liver. WHO's classification – CE3

Patient 20

Female, 61 age

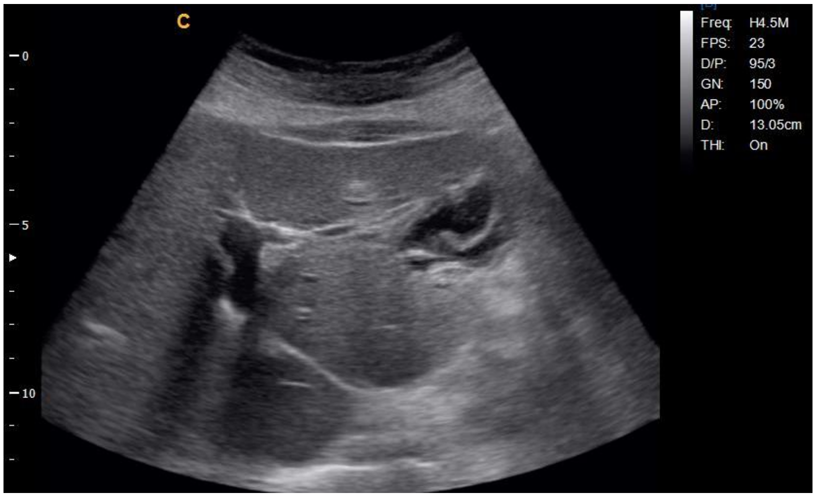

Figure 75: 3.5x3.9 cm, well defined, anechoic, univesicular cyst with detached layers in the liver. WHO's classification – CE3

Patient 21
Male, 70 age

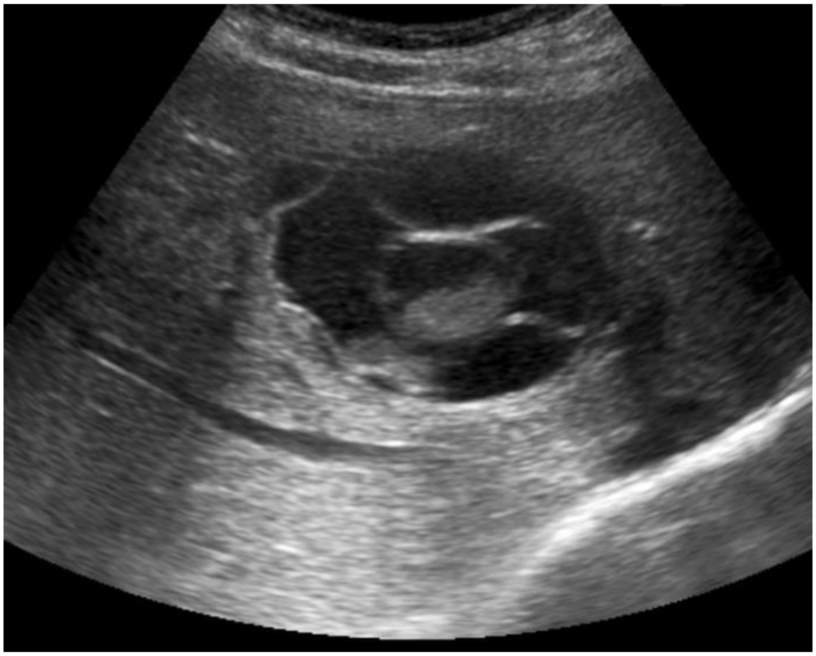

Figure 76: Well defined, multiseptated, anechoic cyst /with double wall sign/ in the liver. WHO's classification – CE2.

Patient 22
Female, 38 age

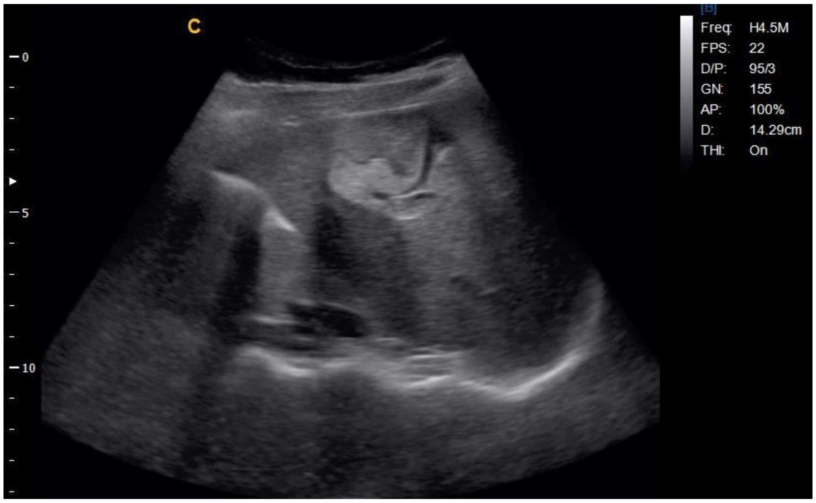

Figure 77: Heterogeneous /hyper and hypo/ echoic mass /with acoustic shadow/ in the liver. WHO's classification – CE4

Patient 23

Male, 62 age

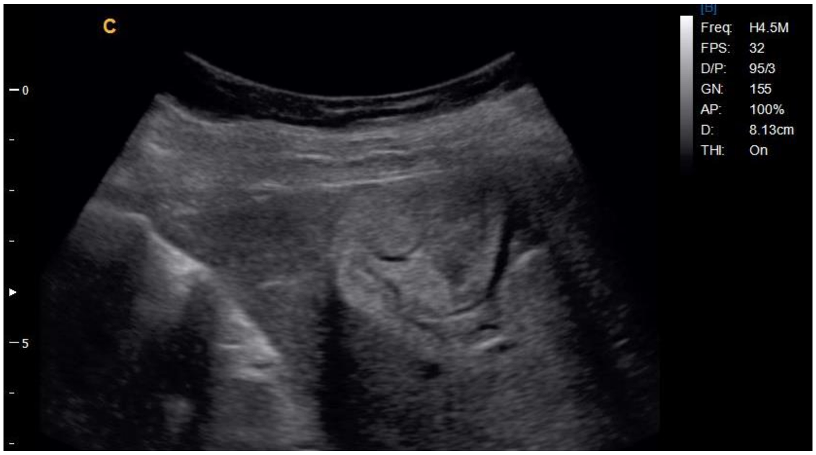

Figure 78: 5.2x4.9 cm, heterogeneous /hyper and hypo/ echoic, cystic with double wall sign in the liver. WHO's classification – CE4

Patient 24

Male, 65 age

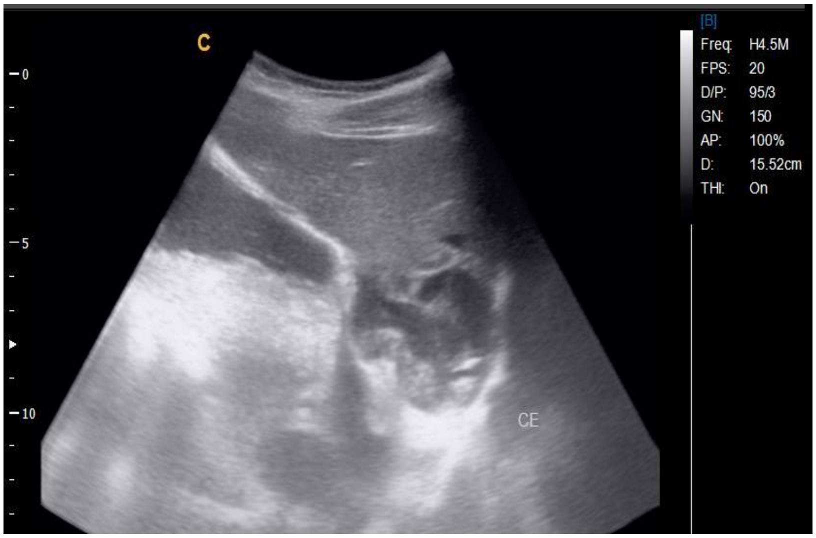

Figure 79: 4.7x4.2 cm, well defined, univesicular cystic with detached layers in the VII segment of the liver. WHO's classification – CE3a

Patient 25

Male, 15 age

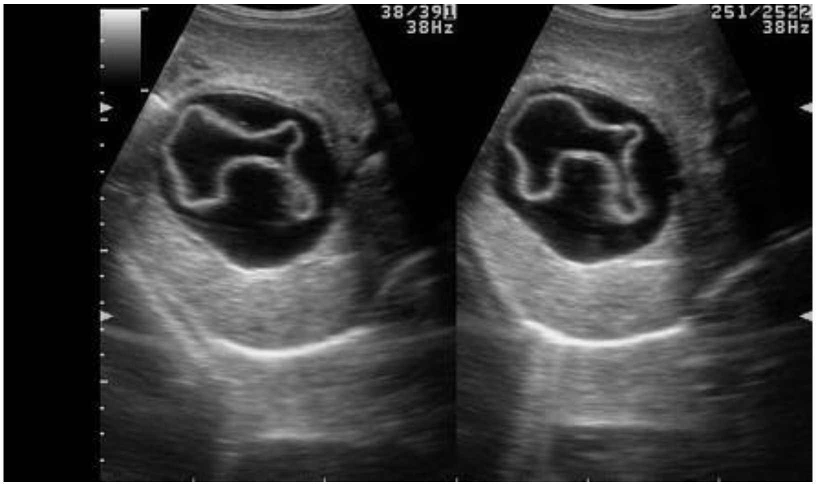

Figure 80: 5.5x5.8 cm, well defined, anechoic, univesicular cyst with detached layers in the liver. WHO’s classification – CE3.

Patient 26

Female, 50 age

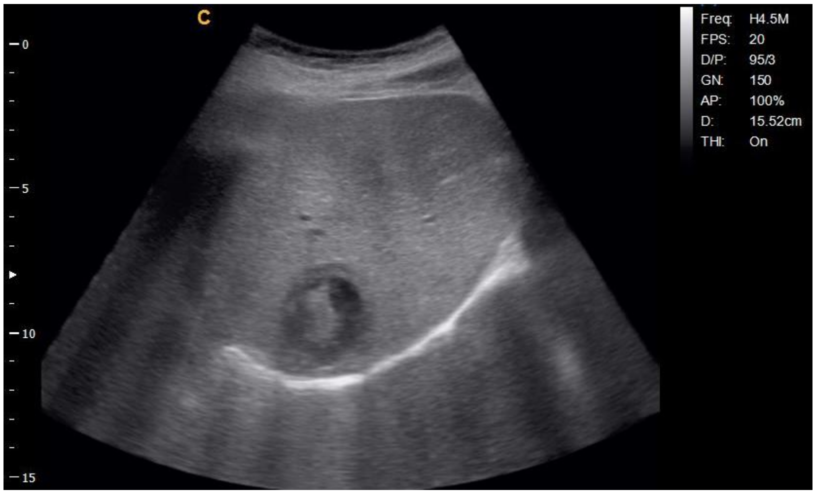

Figure 81: Heterogeneous /hyper and hypo/ echoic, cyst with double wall sign in the liver. WHO’s classification – CE4.

Patient 27

Female, 71 age

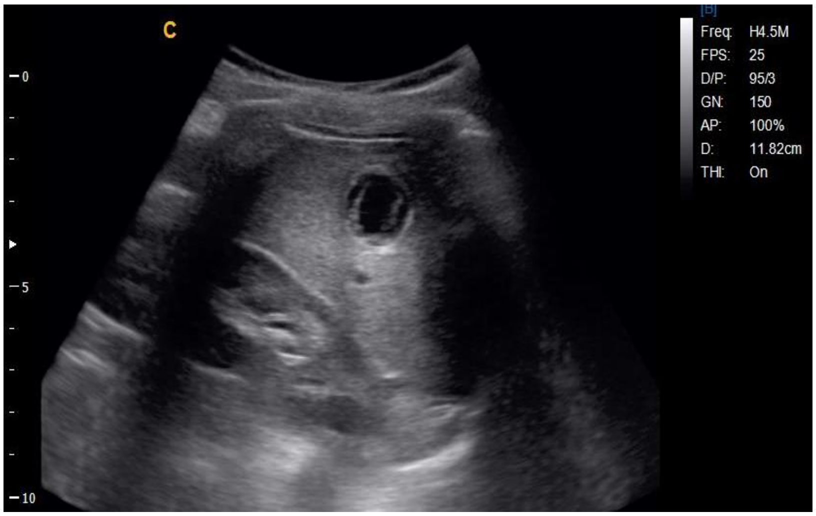

Figure 82: 2.9x3.6 cm, well defined, univesicular cystic with detached layers in the V segment of the liver. WHO’s classification – CE3

ULTRASOUND FIGURES IN PATIENTS OF SUKHBAATAR PROVINCE

Patient 1
Female, 56 age

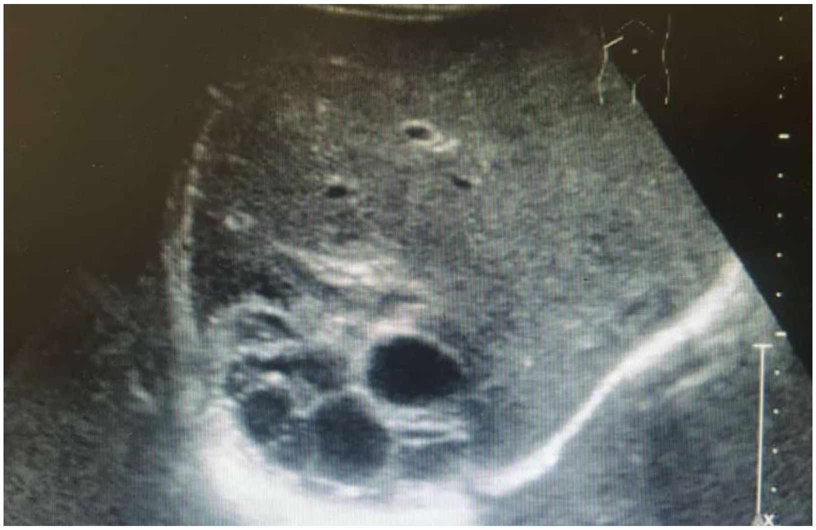

Figure 83: Well defined, multiseptated, anechoic cyst /with double wall sign/ in the right lobe of liver. WHO's classification – CE2

Patient 2
Male, 61 age

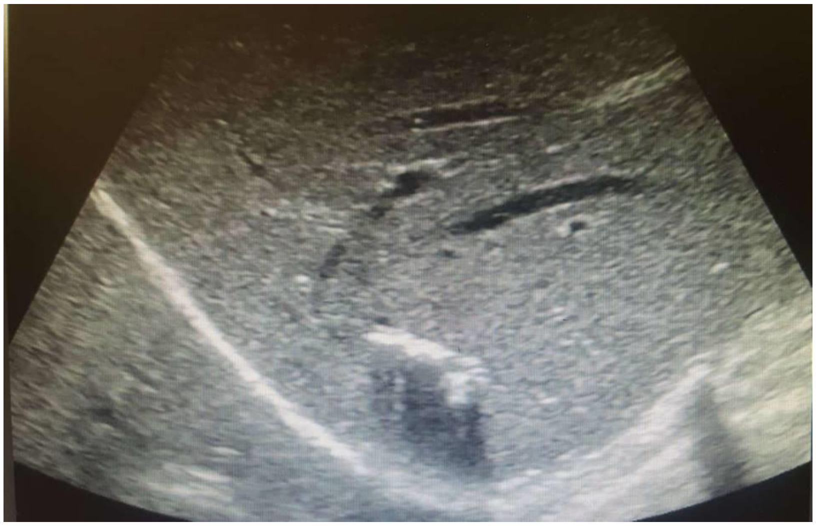

Figure 84: 2.9x3.4 cm, calcified wall with acoustic shadow, solid cyst in the liver. WHO's classification – CE5

Patient 3
Female, 68 age

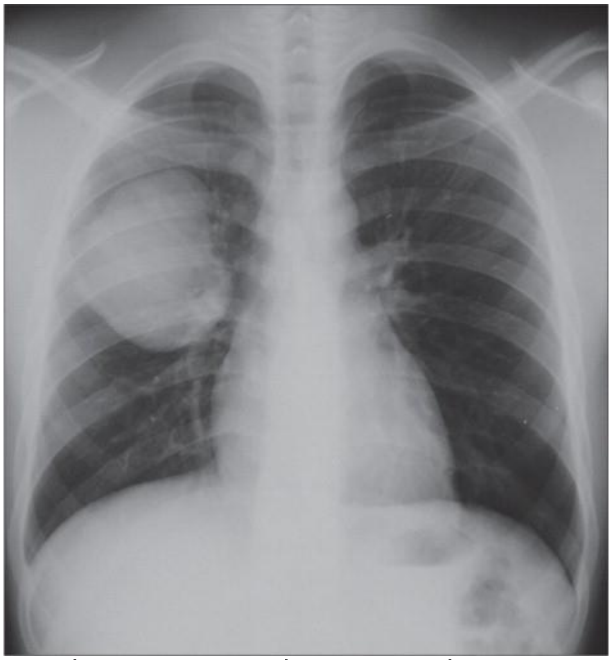

Figure 85: Chest X- ray PA view – Huge, homogeneous focal consolidation in the right middle zone.

Patient 4
Female, 84 age

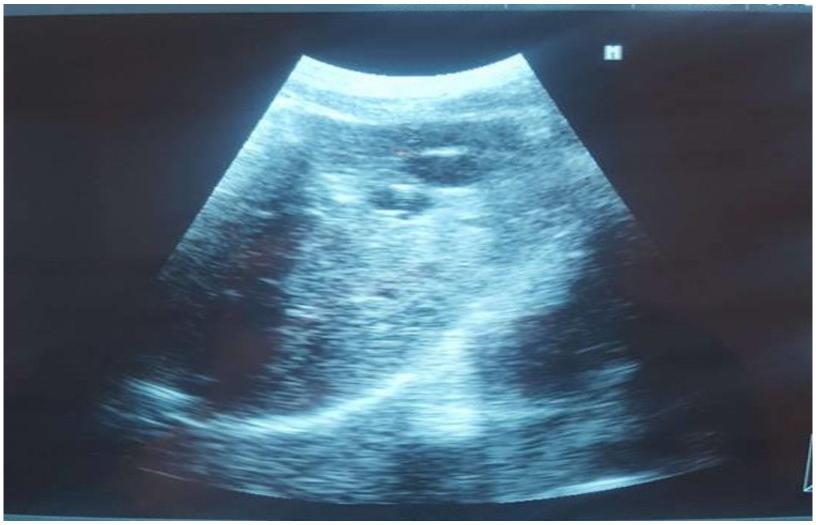

Figure 86: 3.3x3.6cm, well defined, multiseptated, anechoic cyst /with double wall sign/ in the liver. WHO’s classification – CE2

Patient 5

Male, 75 age

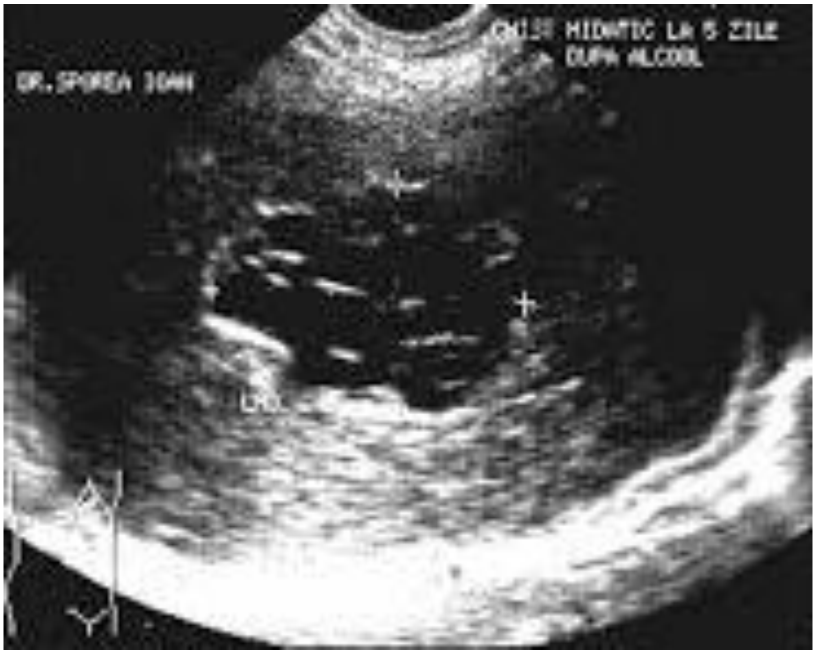

Figure 87: 5.2x5.6 cm, well defined, multiseptated, anechoic cyst /with double wall sign/ in the liver. WHO’s classification – CE2
